# Botulinum Neurotoxins A and E Undergo Retrograde Axonal Transport in Primary Motor Neurons

**DOI:** 10.1371/journal.ppat.1003087

**Published:** 2012-12-27

**Authors:** Laura Restani, Francesco Giribaldi, Maria Manich, Kinga Bercsenyi, Guillermo Menendez, Ornella Rossetto, Matteo Caleo, Giampietro Schiavo

**Affiliations:** 1 Molecular NeuroPathobiology Laboratory, Cancer Research UK London Research Institute, London, United Kingdom; 2 CNR, Neuroscience Institute, Pisa, Italy; 3 Institut Pasteur, Unité des Bactéries anaérobies et Toxines, Paris, France; 4 Department of Biomedical Sciences, University of Padova, Padova, Italy; University of Illinois, United States of America

## Abstract

The striking differences between the clinical symptoms of tetanus and botulism have been ascribed to the different fate of the parental neurotoxins once internalised in motor neurons. Tetanus toxin (TeNT) is known to undergo transcytosis into inhibitory interneurons and block the release of inhibitory neurotransmitters in the spinal cord, causing a spastic paralysis. In contrast, botulinum neurotoxins (BoNTs) block acetylcholine release at the neuromuscular junction, therefore inducing a flaccid paralysis. Whilst overt experimental evidence supports the sorting of TeNT to the axonal retrograde transport pathway, recent findings challenge the established view that BoNT trafficking is restricted to the neuromuscular junction by highlighting central effects caused by these neurotoxins. These results suggest a more complex scenario whereby BoNTs also engage long-range trafficking mechanisms. However, the intracellular pathways underlying this process remain unclear. We sought to fill this gap by using primary motor neurons either in *mass* culture or differentiated in microfluidic devices to directly monitor the endocytosis and axonal transport of full length BoNT/A and BoNT/E and their recombinant binding fragments. We show that BoNT/A and BoNT/E are internalised by spinal cord motor neurons and undergo fast axonal retrograde transport. BoNT/A and BoNT/E are internalised in non-acidic axonal carriers that partially overlap with those containing TeNT, following a process that is largely independent of stimulated synaptic vesicle endo-exocytosis. Following intramuscular injection *in vivo*, BoNT/A and TeNT displayed central effects with a similar time course. Central actions paralleled the peripheral spastic paralysis for TeNT, but lagged behind the onset of flaccid paralysis for BoNT/A. These results suggest that the fast axonal retrograde transport compartment is composed of multifunctional trafficking organelles orchestrating the simultaneous transfer of diverse cargoes from nerve terminals to the soma, and represents a general gateway for the delivery of virulence factors and pathogens to the central nervous system.

## Author Summary

Botulinum neurotoxins are the most toxic molecules known to mankind, and as a result, are currently listed among the top bio-threats. However, their ability to bind specifically to neurons and their inhibitory effects on regulated secretion prompted their clinical use in pathologies characterised by increased muscular tone, such as dystonia and various forms of spasticity, or abnormal secretion, such as drooling and excessive sweating, to cite a few. As a consequence, botulinum neurotoxin A, which is the serotype most commonly used in human therapy, has become the treatment of choice for an ever-expanding number of pathological and non-pathological (e.g. cosmetic) conditions. All current indications show that the systemic effects and toxicity of botulinum neurotoxin A are minimised by the specific route of administration (local injection) and the low diffusion of this molecule in tissues. However, recent reports suggest that in contrast to this common belief, botulinum neurotoxin A is able to reach distal sites in the body and may have previously unanticipated effects in the central nervous system. In this study, we demonstrate that botulinum neurotoxin A and E enter alternative endocytic pathway(s) in addition to synaptic vesicle recycling, and undergo long-range transport in a non degradative compartment in spinal cord motor neurons. Our results show that axonal retrograde transport is a common pathway for the dissemination in the central nervous system of pathogens and virulence factors important for human and animal health.

## Introduction

Through the years, bacterial and animal toxins have been the target of intense medical investigation due to their importance for human health [1]. As such, their structure-function relationships and mechanism of action have been extensively analysed, leading to the development of powerful vaccines and chemical inhibitors targeting either the toxin active site or key events in their cellular intoxication process [2]–[5]. Basic and clinical research into the mechanism of action of these toxins has been boosted by the inclusion of some of them, such as anthrax lethal factor and botulinum neurotoxins (BoNTs), among bioterrorism threats.

In spite of these warfare links, some of these protein toxins have recently acquired novel roles in human medicine, which go beyond their importance as vaccine products. The clearest example is represented by BoNTs, the causal agents of animal and human botulism. BoNTs are large proteins produced by different bacteria of the genus *Clostridia* and together with the closely related tetanus toxin (TeNT) form the clostridial neurotoxin family (CNT) [4], [6], [7]. Seven different serotypes named from A to G (BoNT/A-G) and more than 35 variants are presently known [8]. BoNTs and TeNT are produced as single polypeptides with an average molecular weight of 150 kDa. Single chain BoNTs are converted into fully active neurotoxins by bacterial and tissue proteases, yielding the di-chain fully active molecules. The active form is composed of a heavy (H) chain, in which the carboxy-terminal domain (H_C_) mediates neurospecificity and high affinity binding to receptors present on the plasma membrane of neurons, and a light (L) chain, which is responsible for the intracellular activity of the neurotoxin [4], [6], [9]. The L chain is indeed a zinc-dependent endopeptidase specific for proteins belonging to the SNARE superfamily [10], which have an essential role in the fusion of synaptic vesicles with the pre-synaptic membrane [11]. Cleavage of synaptic SNAREs halts the release of neurotransmitters and is responsible for the long-lasting block of neuroexocytosis observed in cultured neurons [12] and the paralysis elicited by these neurotoxins in vivo [13].

With the exception of BoNT/C, a single substrate has been described for each CNT [6]. BoNT/B, D, F, G and TeNT cleave VAMP, a SNARE protein localised on synaptic vesicles, whilst BoNT/A and E target SNAP25, which is anchored to the plasma membrane at synaptic and extrasynaptic sites. BoNT/C cleaves both SNAP25 and syntaxin, another SNARE localised to the synaptic plasma membrane. BoNTs and TeNT cleave these SNARE proteins at a single peptide bond within their cytoplasmic domain, generating fragments that are unable to sustain membrane fusion and thus neurotransmitter release [10].

Despite their structural and mechanistic similarities, BoNTs and TeNT display striking differences in terms of the type of paralysis induced *in vivo* [5]. BoNTs cause a flaccid paralysis, which is the hallmark of clinical botulism and the basis of a main therapeutic effect observed upon injection of BoNTs in target tissues [7], [10]. This type of paralysis has been attributed to the block of acetylcholine release at the neuromuscular junction, which, as a consequence, is unable to drive contraction of the target muscle [9], [13]. In contrast, TeNT is known to cause a spastic paralysis, which has been attributed to the block in release of inhibitory neurotransmitters in spinal cord interneurons [14]. Several lines of evidence demonstrate that, upon entry into motor neurons, TeNT reaches the spinal cord via long-range axonal retrograde transport [15] followed by transcytosis into neighbouring inhibitory interneurons [16]. Therefore, a different trafficking pathway rather than a diverse intracellular mechanism of action has been suggested to be at the basis of the distinct physiological effects of BoNTs and TeNT.

Several experiments have recently challenged this paradigm demonstrating that BoNT/A is also capable of eliciting its activity in areas distant from the injection site [17]–[20], implying that BoNT/A undergoes long range trafficking in certain experimental conditions.

Here, we show that BoNT/A and BoNT/E are internalised by primary spinal cord motor neurons and undergo fast axonal transport in these cells. BoNT/A and BoNT/E are internalised in non-acidic axonal carriers that contain TeNT, following a process that is largely independent of membrane depolarisation. These results suggest the existence of a long-range transport pathway in motor neurons, which host receptors for several virulence factors and pathogens targeted to the central nervous system.

## Results

### The binding fragments of BoNT/A and BoNT/E are internalised in spinal cord motor neurons

In the last decade, the recombinant binding fragment of TeNT (H_C_T) has been used extensively to monitor internalisation and axonal retrograde transport in many neuronal types [15], allowing the quantitative analysis of these trafficking processes both *in vitro* and in vivo [21]–[24].

**Figure 1:**
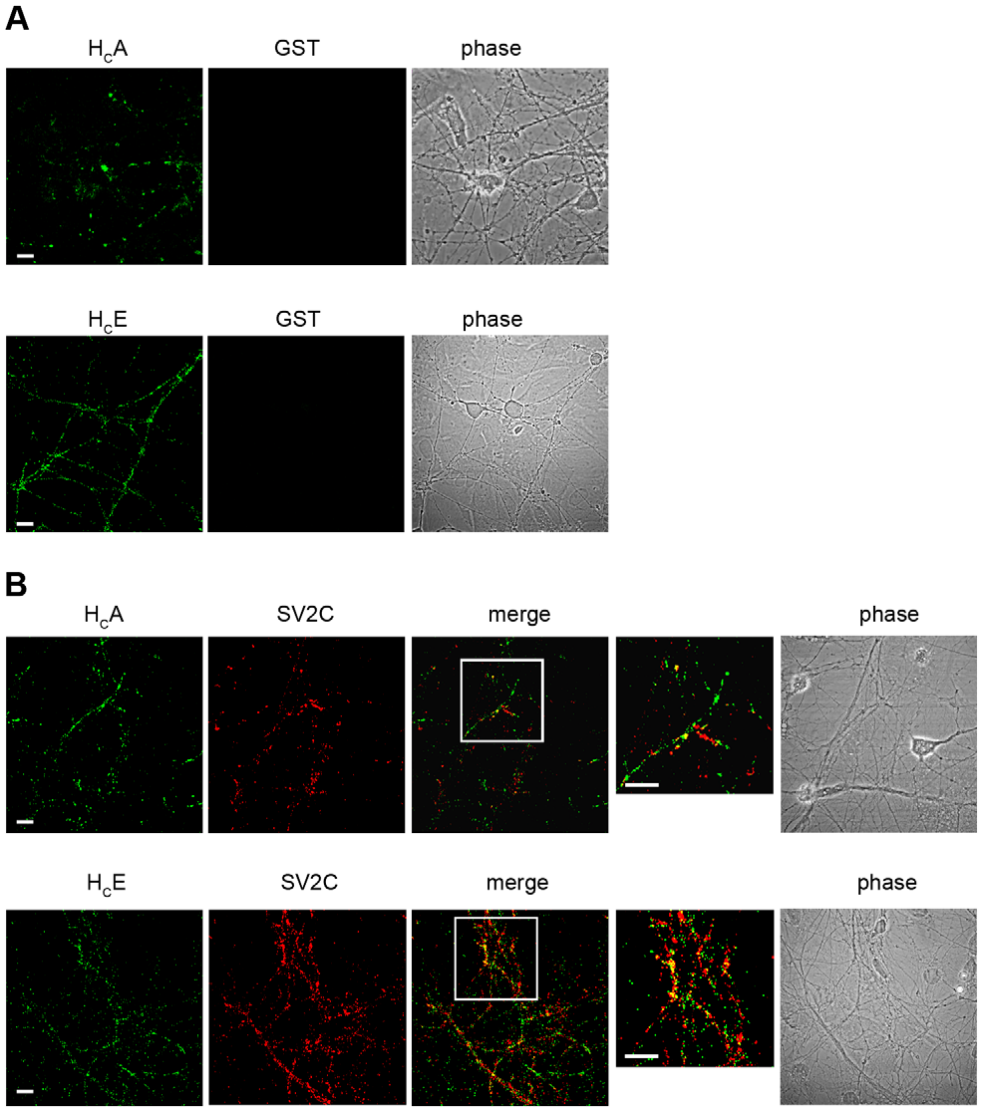
The binding fragments of BoNT/A and BoNT/E bind to the motor neuron surface. (**A**) Motor neurons were incubated with H_C_ fragments for 15 min at 4°C and then fixed. (*top)* Cells were incubated with 15 nM AlexaFluor488-GST-BoNT/A H_C_ (H_C_A) and 15 nM AlexaFluor555-GST (GST) as control. Scale bar, 10 µm. *(bottom)* Motor neurons were incubated with 7.5 nM AlexaFluor488-GST-BoNT/E H_C_ (H_C_E) and 7.5 nM AlexaFluor555-GST (GST) as control. Scale bar, 20 µm. No fluorescence signal was detectable for GST, whilst a punctate signal was present for H_C_A and H_C_E, indicating the ability of these binding fragments to bind to motor neurons. The signal is rather low, as expected in these experimental conditions (4°C). (**B**) Motor neurons were incubated with 15 nM H_C_A *(top)* or 15 nM H_C_E *(bottom)* for 15 min at 4°C, fixed, and stained for SV2C. Only limited colocalisation of H_C_A and H_C_E with SV2C was found in these conditions. Inset: high magnification of the indicated areas. This analysis was performed using two independent primary motor neuron cultures, and replicated at least twice for each motor neuron preparation. Shown are representative images for each condition. Scale bars, 10 µm *(top)*; 20 µm *(bottom)*.

To assess the ability of the binding fragments of BoNT/A (H_C_A) and BoNT/E (H_C_E) to bind and undergo internalisation in living neurons, we expressed them in bacteria as glutathione S-transferase (GST) fusion proteins containing a cysteine-rich tag previously described for H_C_T [21], [25]. Since these GST fusion proteins were significantly more stable than the cleaved products, we decided to use uncleaved GST-H_C_A and GST-H_C_E for our studies. GST tagged with the same cysteine-rich peptide and labelled with a maleimide-based fluorophore was used as a control. To test for binding, spinal cord motor neurons were incubated at 4°C in the presence of fluorescent H_C_A and H_C_E. As shown in Figure 1, a specific signal was detectable on the surface of neurons incubated with H_C_A (15 nM) and H_C_E (7.5 nM), whereas fluorescent GST (15 nM) showed no detectable binding under the same conditions. BoNT/A and BoNT/E have been shown to rely on the synaptic vesicle protein SV2 for their binding and uptake in neurons [26]–[28]. Similarly, preincubation of H_C_A with an excess of a recombinant fragment of SV2C (residues 454–579) fused to GST (1∶100; a kind gift of T. Binz and A. Rummel) compromised the binding and uptake of this fragment in motor neurons (data not shown). We then tested the extent of colocalisation of H_C_A and H_C_E with SV2C using an antibody raised against its cytoplasmic domain. Under the binding conditions used above, only limited colocalisation was however detected between the H_C_A and H_C_E fragments and SV2C on the plasma membrane of resting motor neurons (Figure 1B). The relative low intensity of the signal observed upon incubation of H_C_A and H_C_E at 4°C, and its diffuse nature (Figure 1B) prevented us to perform a reliable quantitative analysis on the extent of colocalisation of these binding fragments with SV2C under our experimental conditions.

**Figure 2:**
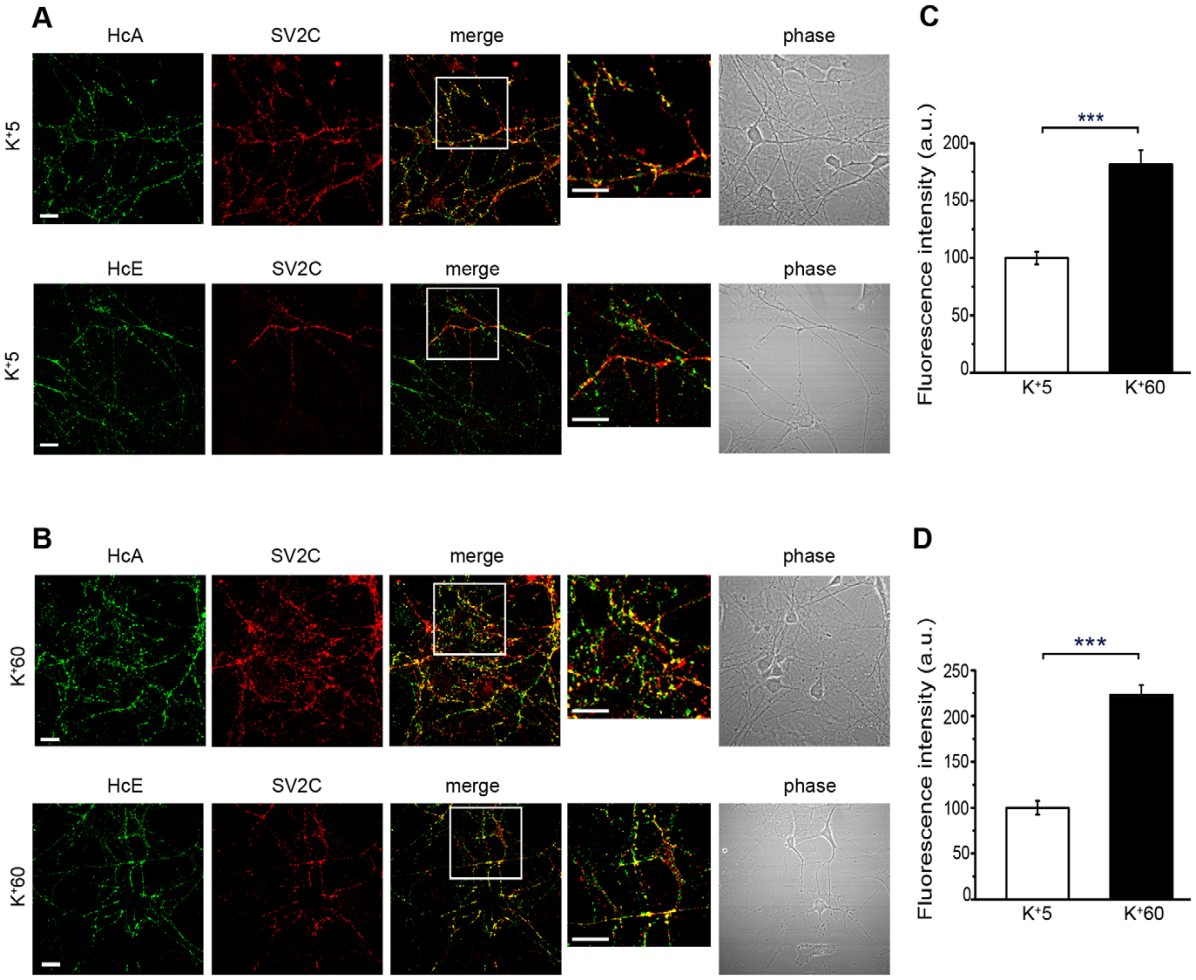
H_C_A and H_C_E are internalised in motor neurons. Motor neurons were incubated with 15 nM H_C_A or 7.5 nM H_C_E for 30 min at 37°C, either under resting conditions (**A**) or after stimulation of synaptic vesicle exo/endocytosis by adding 60 mM KCl to the medium, just before the addition of H_C_s (**B**). Motor neurons were placed on ice, acid washed, fixed, and stained for SV2C. Inset: high magnification of the indicated areas. This analysis was performed using three independent primary motor neuron cultures. Shown are representative images for each condition. Scale bars, 20 µm (A, *top*; B, *top*); 15 µm (A, *bottom*); 40 µm (B, *bottom*). Quantification of the uptake of H_C_A and H_C_E is shown in (**C**) and (**D**), respectively. Bars represent the mean ± standard deviation (SD) of the fluorescence intensity determined from a representative experiment. Ten to thirty fields were analysed for each condition. Although the internalisation of H_C_A (Mann-Whitney test; ***, p<0.001), and H_C_E (Mann-Whitney test; ***, p<0.001), is significantly increased under stimulation, it is extensive also in resting conditions.

BoNT/A and/E, as well as other CNTs, have been shown to interact with both polysialogangliosides and protein receptors on the surface of neuronal cells, and undergo both productive and unproductive binding [29]. To verify whether the binding of H_C_A and H_C_E is compatible with internalisation or is just a dead-end process (e.g. as a result of polysialoganglioside clustering), we incubated motor neurons with labelled H_C_A (15 nM) and H_C_E (7.5 nM) under resting conditions, followed by an acid wash to remove the H_C_ still present on the plasma membrane. When neurons are kept at 4°C, no labelling is detectable upon acid wash (data not shown), as expected for conditions that are known to greatly reduce the rate of endocytosis. However, after incubation for 30 minutes at 37°C, H_C_A and H_C_E puncta resistant to acid stripping, which partially colocalise with SV2, were found in motor neurons (Figure 2A). Interestingly, H_C_A displays similar colocalisation levels with both SV2A and SV2C, whereas H_C_E shows a preference towards SV2C under the same experimental conditions (Mann-Whitney test; **, p<0.01, ***, p<0.001; Figure S1). The finding that both H_C_A and H_C_E are internalised by resting motor neurons and display only a partial colocalisation with SV2 isoforms suggests that these neurotoxins may also exploit an alternative, synaptic vesicle-independent pathway to enter neuronal cells.

Since depolarisation has been shown to promote BoNT/A and/E uptake [30], we then tested whether the endocytosis of H_C_A and H_C_E is enhanced by depolarising motor neurons with 60 mM KCl (Figure 2B). Under these conditions, internalisation occurs with higher efficiency for both H_C_A and H_C_E, resulting in a statistically significant increase (Mann-Whitney test; ***, p<0.001; Figure 2C, D). However, the colocalisation observed between the H_C_ fragments and SV2A and C does not significantly increase under depolarising conditions (Figure 2B and Figure S1), suggesting that H_C_A and H_C_E do not only enter SV2-positive synaptic vesicles but also other endocytic organelles in primary motor neurons.

This unexpected behaviour was not due to the recombinant H_C_s, since similar results were obtained using full length BoNT/A and BoNT/E. In this case, the depolarising conditions seem even less efficient in promoting the internalisation of the full length toxins and enhancing the colocalisation with SV2C (Figure S2).

### BoNT/A and/E are internalised in motor neurons by multiple endocytic routes

**Figure 3:**
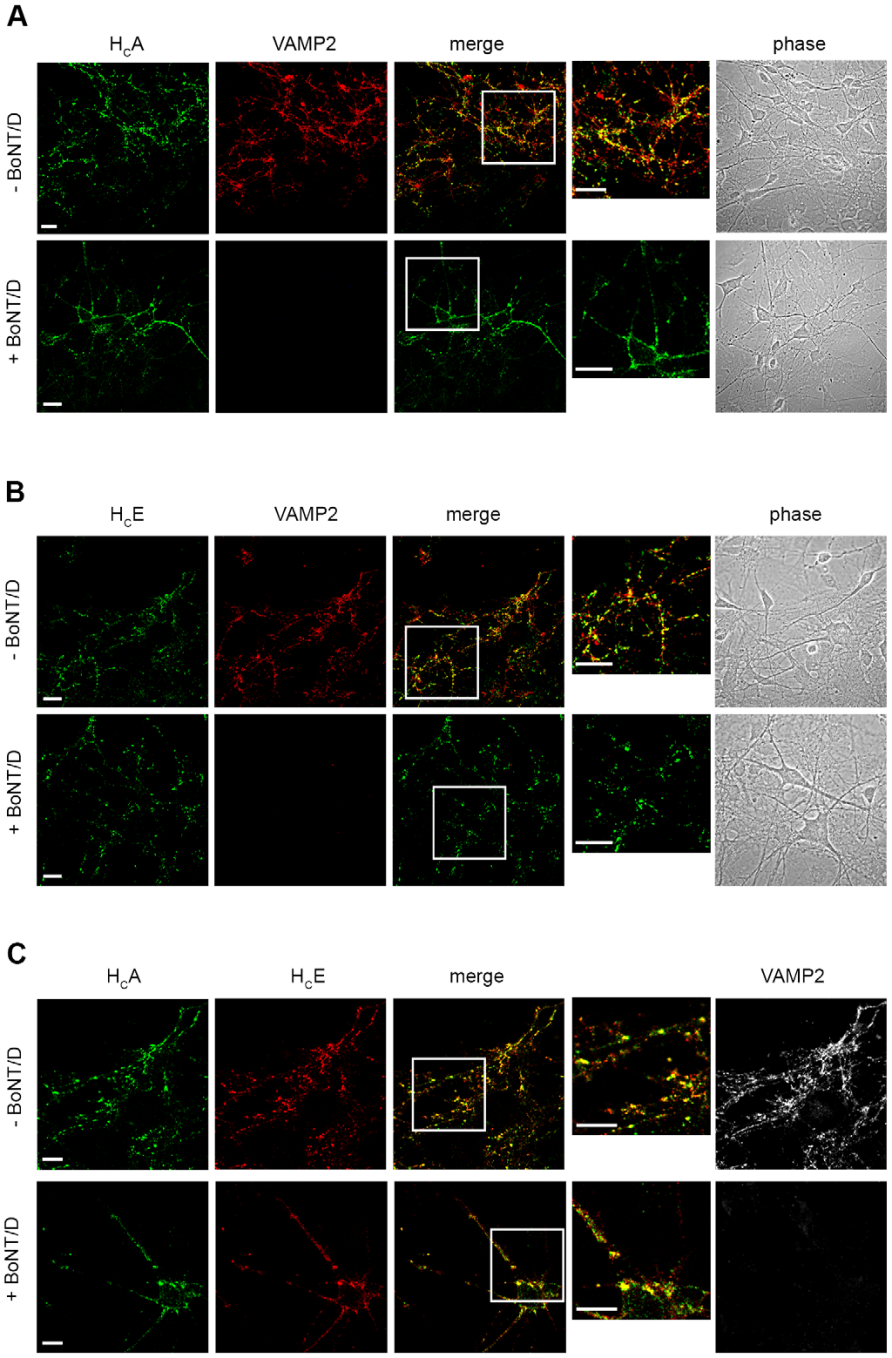
The internalisation of H_C_A and H_C_E in motor neurons occurs in absence of synaptic vesicle exocytosis and recycling. Motor neurons were incubated with 15 nM H_C_A (**A**) or 7.5 nM H_C_E (**B**) for 30 min at 37°C, under stimulating conditions (60 mM KCl). Cells were then placed on ice, acid washed, fixed, and stained for VAMP2. Inset: high magnification of the indicated areas. (A, B, C; *bottom panels*) Motor neurons were pre-treated with 2 nM BoNT/D to cleave VAMP2, thus blocking synaptic vesicle exo/endocytosis. Treatment with BoNT/D did not prevent internalization of H_C_A and H_C_E. (**C**) The colocalisation of H_C_A and H_C_E is largely independent of BoNT/D treatment. The results reported in this figure are representative of experiments performed using two independent primary motor neuron cultures. Scale bars, 20 µm (A, *top and bottom*; B, *top*); 10 µm (B, *bottom*); 5 µm (C).

To further investigate the role of the synaptic vesicle cycle in the internalisation of H_C_A and H_C_E, we took advantage of the specific inhibition caused by BoNT/D on synaptic vesicle exocytosis. BoNT/D blocks the fusion of synaptic vesicles and their recycling by cleaving VAMP. We pre-incubated motor neurons with medium alone or containing 2 nM BoNT/D for 22 h, followed by the addition of H_C_A and H_C_E under depolarising conditions (60 mM KCl). As shown in Figure 3A and B, pre-treatment with BoNT/D did not prevent the internalisation of H_C_A and H_C_E, in spite of the complete cleavage of VAMP2 in treated neurons. Consistent with the results shown above, the colocalisation observed between H_C_A, H_C_E and VAMP2 at synaptic sites is incomplete (Figure 3A, B; upper panels), but qualitatively similar to that detected with SV2C and SV2A (Figure 2 and Figure S1), suggesting that these neurotoxins exploit multiple routes for their entry into motor neurons [31]. These routes extensively overlap for H_C_A and H_C_E, since these two binding fragments displayed an almost complete colocalisation when internalised together in cultured motor neurons under depolarising conditions in the presence or absence of BoNT/D (Figure 3C).

Taken together, these experiments demonstrate that H_C_A and H_C_E are reliable tools to monitor the binding and uptake of their parental neurotoxins in primary motor neurons. Furthermore, the limited effect of BoNT/D on H_C_A and H_C_E internalisation provides a very good qualitative indication that synaptic vesicle endo-exocytosis is not the only mechanism responsible for the uptake of these binding fragments in motor neurons and that an endocytic pathway(s) largely independent of synaptic vesicle recycling is involved in BoNT/A and/E uptake in both resting and depolarising conditions.

### H_C_A and H_C_E undergo retrograde transport in motor neurons

Our observation that the binding fragments of BoNT/A and/E enter motor neurons by parallel endocytic routes begs the question as to the fate of the organelles containing H_C_A and H_C_E. Whereas synaptic vesicles are known to recycle within the synapse and only occasionally transfer inter-synaptically [32], [33], other endocytic compartments are targeted to distal sites via long-range transport pathways. Endogenous ligands, such as neurotrophins and their receptors, lectins (e.g. wheat germ agglutinin) and pathogens (e.g. several neurotropic viruses) enter axonal carriers that undergo microtubule-dependent retrograde transport to the cell body [15]. Since H_C_T is an established probe for this trafficking pathway [21]–[24], we sought to test whether BoNT H_C_s share the same axonal compartment as H_C_T.

**Figure 4:**
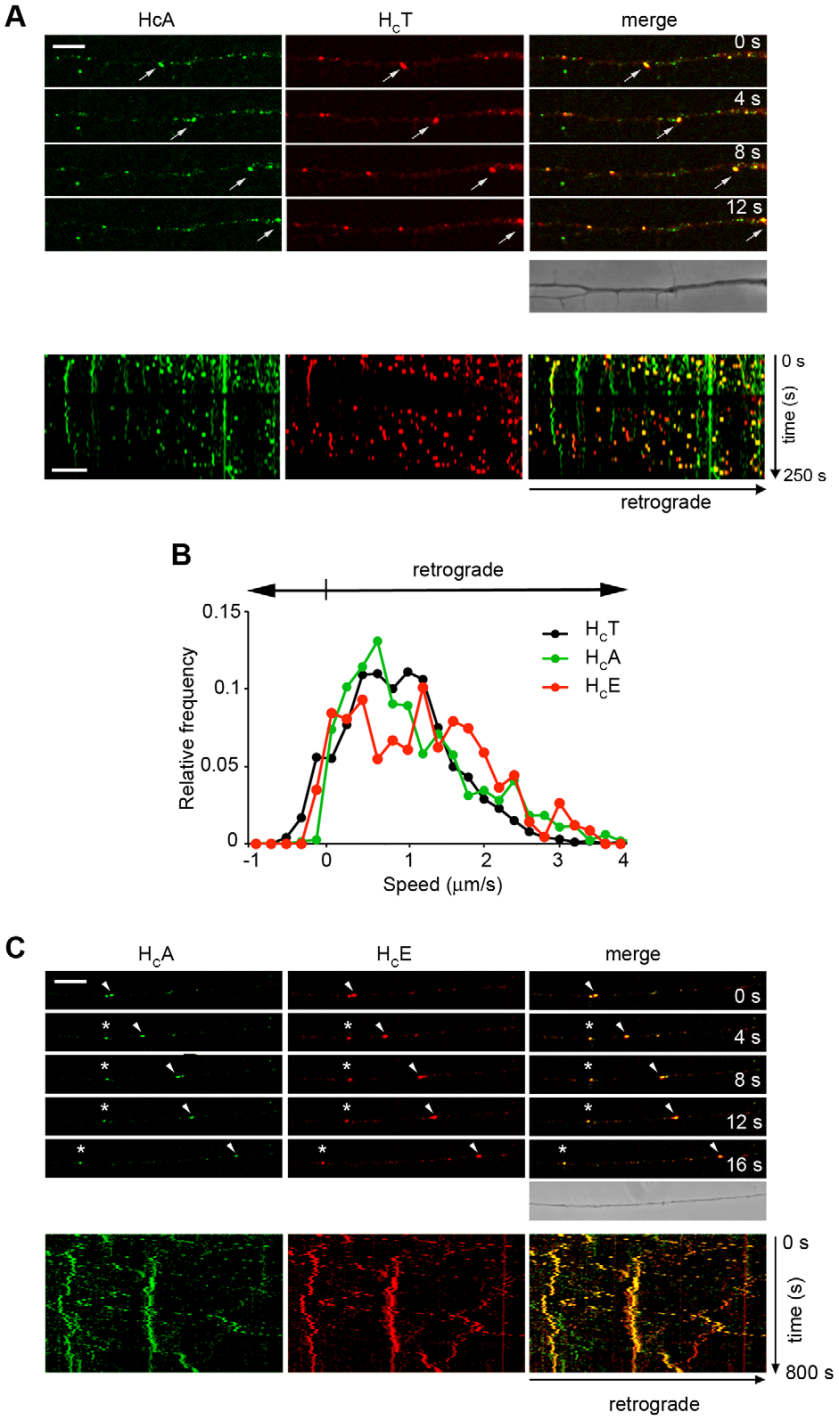
H_C_A, H_C_E and H_C_T share axonal retrograde carriers. Motor neurons were incubated with 15 nM H_C_A and 40 nM AlexaFluor555-TeNT H_C_ (H_C_T) for 30 min at 37°C, either under resting (5 mM KCl) or stimulating conditions (60 mM KCl; data not shown), and then imaged. (**A**, *top*) Individual frames from a confocal time series are shown. Cell bodies are located out of view on the right. Arrows point to double-positive carriers for H_C_A and H_C_T moving towards the soma. Scale bar, 10 µm. (**A**, *bottom*) Kymographs of motor neuron axons treated as described above. The soma is out of view on the right. H_C_T-positive carriers are frequent and fast, whilst several H_C_A-positive organelles are stationary or oscillate. Carriers containing only H_C_A or H_C_T are also present. (**B**) Speed profile of H_C_A (in green), H_C_E- (in red) and H_C_T (in black) carriers. Note the overlap between the three curves, which display a speed peak around 0.8–1 µm/s. (**C**) Motor neurons were incubated with 15 nM H_C_A and 7.5 nM H_C_E for 30 min at 37°C under stimulating conditions (60 mM KCl), and then imaged. (**C**, *top*) Individual frames from a confocal time series are shown. Cell bodies are located out of view on the right. Arrowheads point to a carrier moving towards the soma containing both H_C_A and H_C_E. Asterisks indicate an example of stationary double-positive carrier. Scale bar, 10 µm. (**C**, *bottom*) Kymographs of motor neuron axons correspond to the stills above. Note the high number of double-positive carriers, either moving towards the soma or oscillatory. This analysis was performed using three (H_C_A, H_C_T) or two (H_C_E) independent primary motor neuron cultures, and replicated at least twice. At least ten (H_C_A, H_C_T) or five (H_C_E) movies per conditions were used to assemble the speed distribution curves shown in (**B**).

We incubated motor neurons with 15 nM H_C_A and 40 nM H_C_T for 30 min at 37°C either in resting (5 mM KCl) or depolarising (60 mM KCl) conditions prior to shifting them on ice and acid wash. The latter two treatments were omitted when live imaging was performed. As shown in Figure S3, there was a good colocalisation between H_C_A and H_C_T in resting and depolarised neurons. Interestingly, a fraction of these H_C_A- and H_C_T-positive axonal puncta were mobile and underwent fast retrograde transport towards the soma (Figure 4A). Colocalisation was not limited to moving carriers, but was also frequently observed at the level of stationary organelles (data not shown). Quantitative analysis of the retrograde transport of H_C_A displays a multimodal kinetics with an average speed of 0.8 µm/s (Figure 4B). Interestingly, the speed distribution profiles of H_C_A and H_C_T largely overlapped, indicating, together with their colocalisation in axons (Figure 4A) that these two binding fragments are transported by the same class of axonal organelles.

Whereas distal effects of BoNT/A have been described in multiple systems [17], [20], [34], such evidence for BoNT/E has been lacking [20]. Our finding that H_C_A undergoes retrograde transport in neurons raises the possibility that the different physiological effects of BoNT/A and/E are due to their differential ability to be recruited to this transport route once internalised in motor neurons. Therefore, we performed comparative assays to better understand whether the similarity between these two neurotoxins is only limited to binding and internalisation or if it extends to axonal transport.

Motor neurons were incubated with H_C_A and H_C_E and analysed by time-lapse confocal microscopy (Figure 4C). Strikingly, the extensive colocalisation previously found between H_C_A and H_C_E was not only limited to stationary structures, but also involved organelles transported in the retrograde direction (Figure 4C). Kinetic analysis of H_C_E transport revealed a mainly retrograde speed distribution profile, which overlaps with those of H_C_A and H_C_T (Figure 4B).

### H_C_A undergoes axonal retrograde transport in compartmented motor neuron cultures

**Figure 5:**
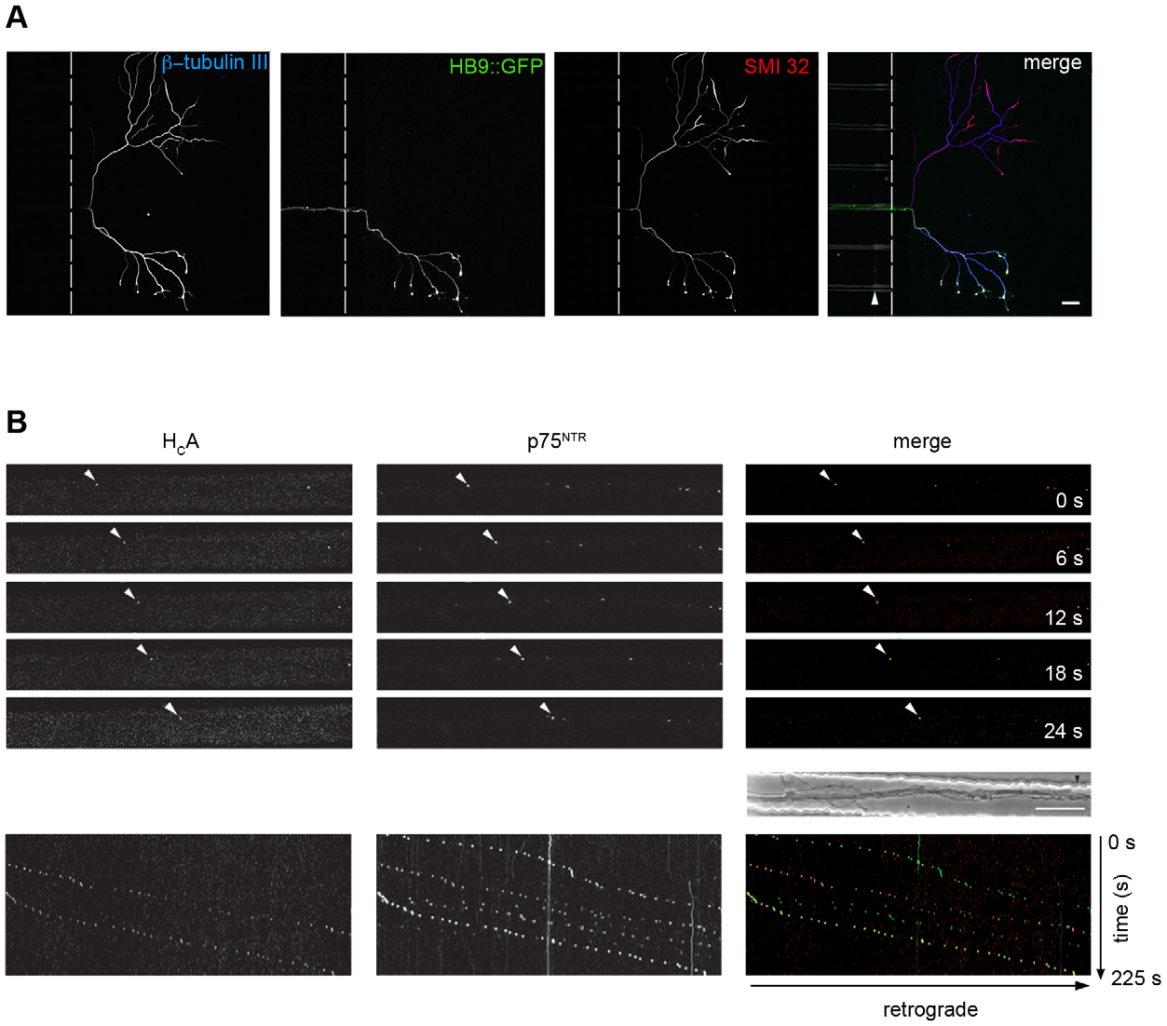
H_C_A undergoes axonal retrograde transport with the neurotrophin receptor p75^NTR^ in microfluidic chambers. (**A**) Axonal compartment of a MFC. Motor neurons differentiated from an embryonic stem (ES) cell line expressing GFP under the control of the motor neuron-specific promoter (HB9::GFP) have been seeded in the somatic compartment (on the left; not shown). Upon differentiation, their axons elongated through the microgrooves (arrow in the *merge* panel) and reached the axonal compartment (hatched lines indicate the boundary of this compartment). Cells were stained for the neuronal marker βIII tubulin (blue) and the axonal marker SMI32 (red). Cell bodies are out of view on the left. Scale bar, 50 µm. (**B**) Primary mouse motor neuron cultures in MFC were used to assess the axonal retrograde transport of H_C_A. Motor neurons were incubated with fluorescent H_C_A (red, on the left) and with a fluorescent antibody for p75^NTR^ (green, middle), for 30 min at 37°C in resting conditions, then washed and imaged. Both H_C_A and the antibody have been added to the axonal side only of the MFC. Representative stills from a confocal time series are shown in the top part of the panel whilst the relative kymographs are shown at the bottom. Arrowheads indicate double-positive retrogradely transported organelles. Cell bodies are located out of view on the right. The analysis shown in (**B**) is representative of two independent primary motor neuron cultures. Scale bar, 20 µm.

The experiments presented so far were performed using spinal cord preparations enriched in motor neurons in *mass* cultures. Under these conditions, it is not always possible to provide an unequivocal identification of the type of neuron imaged at any given time, nor to assess the site of internalisation of the neurotoxin added to the medium. To overcome these technical shortcomings, we exploited a compartmentalised system based on microfluidic chambers (MFC), which allows the separation of cell bodies from axon terminals and is suitable for live imaging [35], [36] and biochemical analyses [36]. To avoid diffusion between axonal and cell body compartments, the volume of medium in the latter compartment was maintained at higher levels at all times to ensure a laminar flow towards the axonal side. In a first set of experiments, we used as a source of motor neurons, an embryonic stem (ES) cell line stably transfected with a construct encoding green fluorescent protein (GFP) under the control of a motor neuron-specific promoter (HB9::GFP) [37]. Motor neurons differentiated from this ES cell line express GFP in their cytoplasm, allowing their unambiguous identification. Cells were also counterstained with the pan-neuronal marker βIII tubulin and the axonal marker SMI32. An example of a motor neuron axon positive for βIII tubulin, SMI32 and GFP, crossing the microgroove of the MFC is shown in the lower part of Figure 5A, whilst an axon belonging to a different type of neuron (or a motor neuron in which the HB9 promoter has already been switched off) not expressing GFP is visible in the upper part of the same panel.

To further prove that the axonal transport of H_C_A seen in *mass* cultures occurs in a retrograde direction, we incubated mouse primary motor neurons with fluorescently-labelled H_C_A alone (Movie S1) or together with an antibody directed against the neurotrophin receptor p75^NTR^ for 30 minutes at 37°C, adding the two probes only to the axonal side of the MFC. We then monitored axonal transport in the MFC microgrooves using time-lapse microscopy. Representative stills of a movie (Movie S2) displaying retrogradely transported H_C_A and p75^NTR^positive organelles moving retrogradely are shown in Figure 5B, together with the corresponding kymograph. Interestingly, several of the axonal carriers containing H_C_A are also positive for the neurotrophin receptor p75^NTR^, an established marker of the axonal retrograde transport compartment [23], [38]–[40]. Altogether, these findings demonstrate that in motor neurons H_C_A and H_C_E undergo fast axonal retrograde transport in endosomal carriers, which are shared with H_C_T and endogenous cargoes, such as the neurotrophin receptor p75^NTR^.

### H_C_A and H_C_E are transported in non-acidic transport carriers

These results prompted us to further characterise the moving organelles containing H_C_A and H_C_E. Entry of TeNT and BoNTs in acidic compartments is indeed required for the translocation of the L chain into the cytoplasm and cleavage of its SNARE target with the resulting inhibition of neurotransmitter release [41].

**Figure 6:**
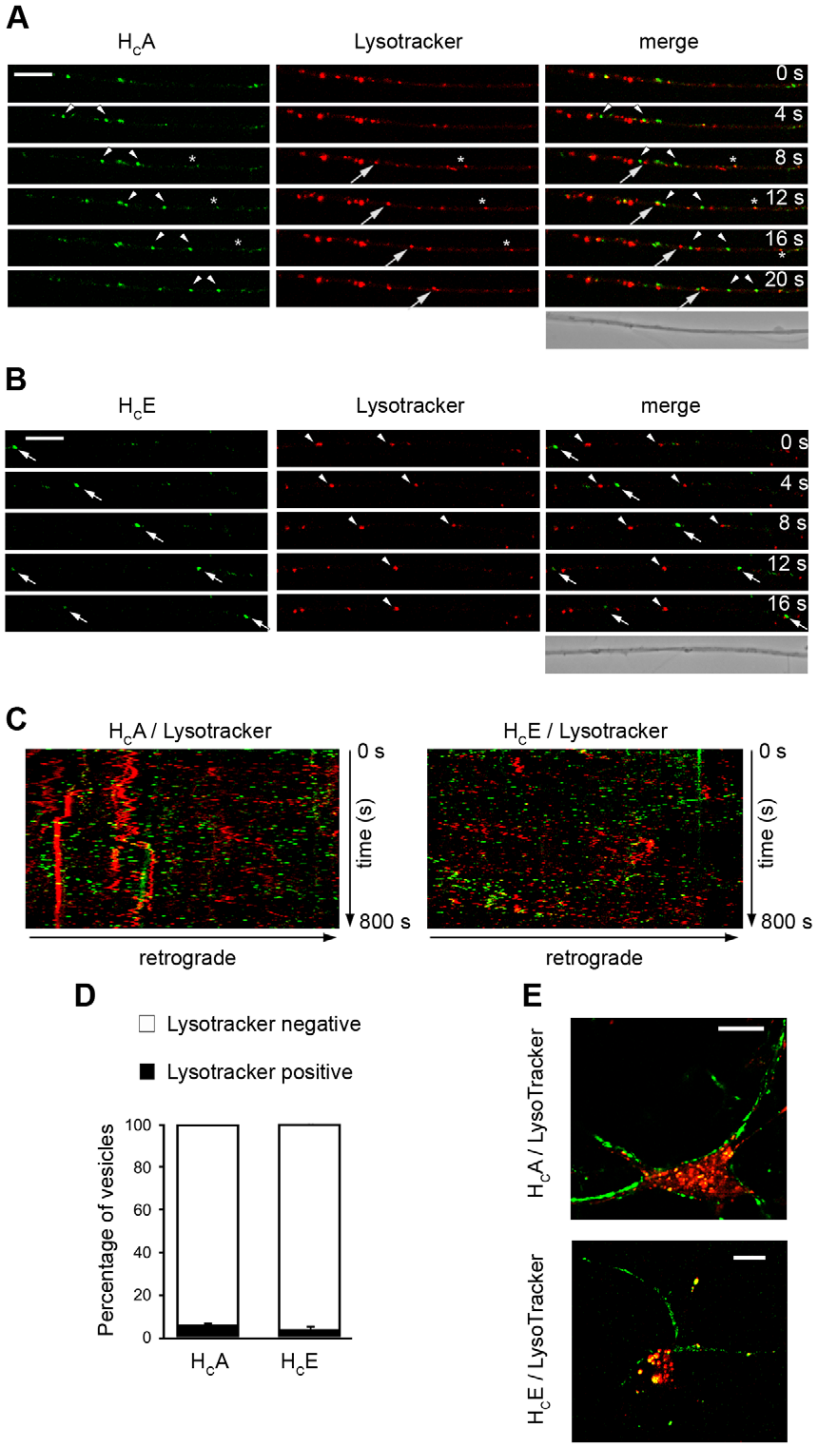
H_C_A- and H_C_E-positive carriers do not colocalise with acidic organelles. Motor neurons were incubated with 15 nM H_C_A (**A**) or 7.5 nM H_C_E (**B**) and 50 nM Lysotracker Red for 30 min at 37°C, under stimulatory conditions. Cells were then washed and imaged. (**A**) Individual frames from a confocal time series are shown. Cell bodies are located out of view on the right. Arrowheads mark H_C_A-positive carriers, whilst arrows point out Lysotracker-positive organelles. Asterisks indicate a small structure containing both H_C_A and Lysotracker. Scale bar, 10 µm. (**B**) Individual frames from a confocal time series are shown. Cell bodies are located out of view on the right. Arrows mark H_C_E-positive carriers, whilst arrowheads point out Lysotracker-positive organelles. Scale bars, 10 µm. (**C**) Kymographs correspondent to the time series described above. The graphs resulted from the merge between H_C_A (in green; left) or H_C_E (in green; right) and Lysotracker (in red). Note the virtual absence of double-positive organelles. (**D**) Quantification of H_C_-Lysotracker carriers. The percentage of double-positive structures is very low, indicating that H_C_A and H_C_E were transported in non-acidic organelles. Bars represent the mean ± standard deviation (SD) and were obtained by analysing at least five movies per condition. (**E**) Negligible colocalisation between H_C_A or H_C_E and Lysotracker is also observed in the soma. Scale bars, 10 µm. The results reported in this study were obtained using two independent primary motor neuron cultures, and replicated twice per condition.

To test the presence of H_C_A and H_C_E in acidic carriers, we incubated motor neurons cultures with H_C_A and H_C_E together with Lysotracker, a probe that accumulates only in acidic vesicles. Neither H_C_A (Figure 6A; Movie S3), or H_C_E (Figure 6B; Movie S4) were found in Lysotracker-positive organelles in the axons of motor neurons. This result was confirmed by the almost complete absence of yellow organelles in the kymographs corresponding to H_C_A (Figure 6C; left panel) and H_C_E (Figure 6C; right panel) and quantified in Figure 6D (H_C_A-Lysotracker positive carriers, 5.2±2.0%; H_C_E-Lysotracker positive carriers, 3.0±3.0%). The overlap between H_C_A- and H_C_E-positive compartments with acidic organelles was very limited in the soma as well (Figure 6E), indicating that H_C_A and H_C_E are transported and sorted in non-acidic organelles in spinal cord motor neurons.

### Full length BoNT/A and BoNT/E enter retrograde transport carriers with different efficiency

**Figure 7:**
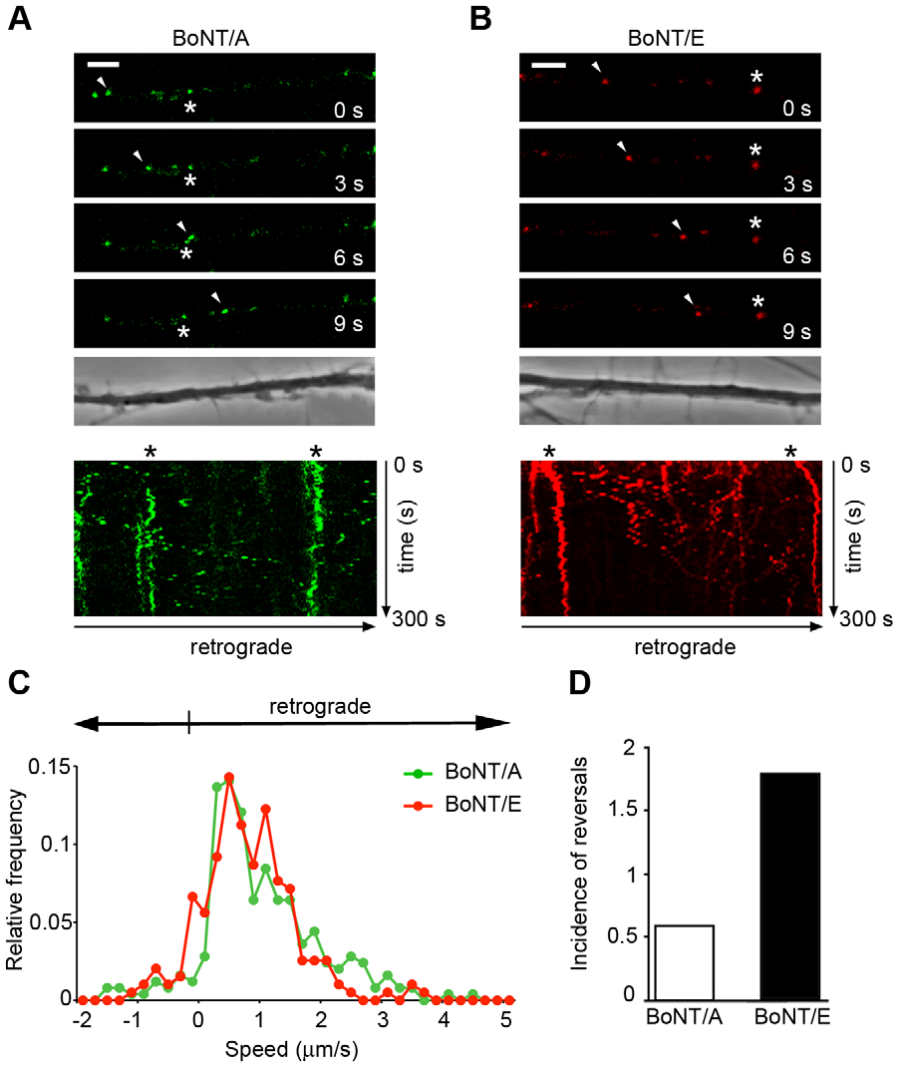
Full length BoNT/A and BoNT/E undergo retrograde transport in motor neurons. Motor neurons were incubated with 30 nM full length AlexaFluor488-BoNT/A (**A**) or 30 nM full length AlexaFluor555-BoNT/E (**B**) for 30 min at 37°C in resting conditions, then washed and imaged. (**A**, *top*) Individual frames from a confocal time series are shown. Cell bodies are located out of view on the right. Arrowheads mark a BoNT/A-positive carrier moving towards the soma. Asterisks indicate an example of stationary carrier. (**A**, *bottom*) Kymograph corresponding to the time series described above. Scale bar, 5 µm. (**B**, *top*) Individual frames from a confocal time series are shown. Cell bodies are located out of view on the right. Arrowheads mark a BoNT/E-positive carrier moving towards the soma. Asterisks indicate an example of a stationary carrier. (**B**, *bottom*) Kymograph corresponding to the time series described above. Scale bar, 5 µm. (**C**) Speed distribution profile of full length BoNT/A (in green) or BoNT/E (in red) carriers. Note the similar transport kinetics of the two neurotoxins. (**D**) The incidence of reversals (number of changes of direction per organelle) for BoNT/E carriers is threefold higher than for BoNT/A-positive organelles. Data were obtained by analysing at least ten movies for each neurotoxin. The results were confirmed in two independent primary motor neuron cultures, and replicated twice per condition.

Prompted by the results obtained with H_C_A and H_C_E, we tested the ability of the full-length neurotoxins to undergo retrograde transport. To this end, spinal cord motor neurons were incubated with full length fluorescent BoNT/A or BoNT/E and imaged using confocal time-lapse microscopy. Individual frames from a time series clearly showed that both BoNT/A (Figure 7A) and BoNT/E (Figure 7B) are transported in axons. Kymographs derived from these stills indicated the presence of both stationary and moving organelles with different speed and directions (Figure 7A and B; lower panels).

Quantitative kinetic analyses revealed similar speed distribution profiles for full length BoNT/A and BoNT/E, with a slight increase in the frequency of pauses and movements in the anterograde direction for the latter (Figure 7C). Although both neurotoxins are retrogradely transported, this process seems to occur with different modalities for BoNT/A and/E. Whereas fast retrogradely-transported organelles showing progressive movements were detected with full length BoNT/A, BoNT/E-positive carriers displayed a less continuous motion toward the cell body, as demonstrated by a higher frequency of reversals (Figure 7D). These transitory changes in direction of the carriers could account for the presence of a peak at −0.2 µm/s (anterograde direction) in the speed distribution profile of BoNT/E and a higher frequency of pauses (Figure 7D). These differences in the transport kinetics of BoNT/A and BoNT/E might contribute to the lower efficiency of BoNT/E in cleaving SNAP25 in cell bodies compared to BoNT/A, when these neurotoxins are applied to axons in compartmentalised cultures of sympathetic neurons [18]. Altogether, these results suggest that full length BoNT/A and/E are retrogradely transported in primary motor neurons, albeit with different efficiency.

### Long-distance effects of BoNT/A in spinal cord motor neurons

The next goal of our study was determining whether these neurotoxins reach the soma in an active form, since this would provide mechanistic insights on their long-range mode of action *in vivo*. We assessed the long-range effects of BoNT/A by monitoring the appearance of the cleaved fragment of SNAP25, which can be distinguished from the full-length protein using an antibody specific for the cleaved form [17], [19], [20], [34].

**Figure 8:**
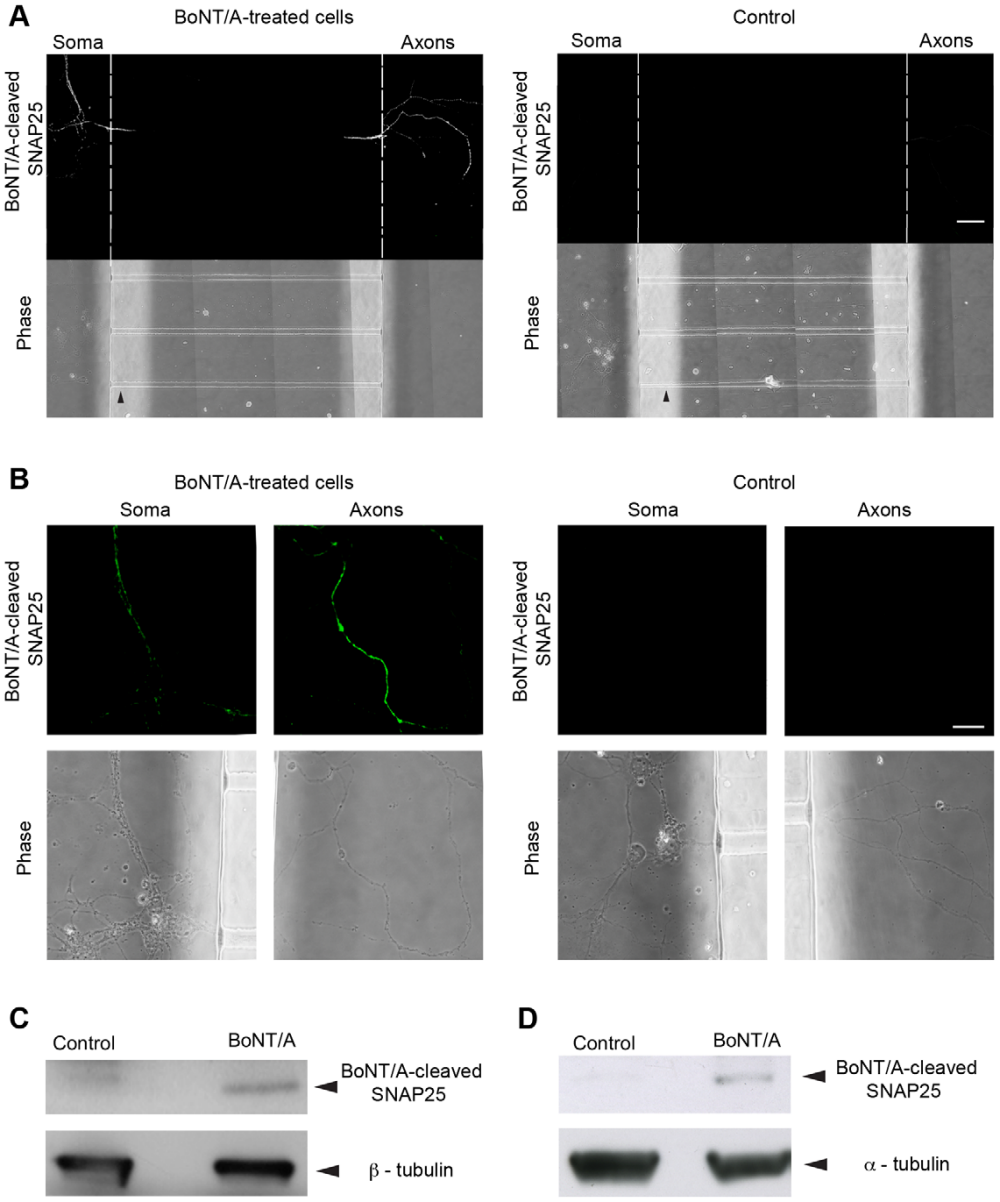
Distal effects of BoNT/A. Long-range effects of BoNT/A were assessed by immunofluorescence (**A, B**) and western blot, either *in vitro* (**A**–**C**) or *in vivo* (**D**), using an antibody that specifically recognises the fragment of SNAP25 generated by BoNT/A. Motor neurons grown in MFC were incubated with 10 nM BoNT/A added to the axonal compartment only of MFC. Samples were processed as described in Experimental Procedures. (**A**) Tile scan of a representative MFC treated with BoNT/A (left) and the untreated control (right). Arrowheads indicate the microgrooves whilst the hatched lines indicate the boundaries between the cell body and the axonal side of the MFC. Scale bar, 50 µm. (**B**) High magnification images of MFCs treated as in **A**. Controls were run in parallel and the microscope settings used for image acquisition were kept constant. Scale bar, 10 µm. Similar results were obtained using two independent primary motor neuron cultures, and replicated twice per condition. (**C**) Western blot revealed the presence of BoNT/A-cleaved SNAP25 (24 kDa) in the somatic compartment of MFC treated only on the axonal side with full length BoNT/A. No signal was present in control MFCs. This result demonstrates that BoNT/A undergoes axonal retrograde transport in a fully active form and was confirmed in two independent experiments. A representative western blot is shown in (**C**). β-tubulin (50 kDa) was used as loading control. (**D**) Representative western blot for BoNT/A-cleaved SNAP25 on extracts from lumbar spinal cord segments of a naive (control) animal and a rat injected with BoNT/A into the hind limb muscles 10 d earlier. These results were confirmed in two independent experiments, replicated three times. A representative western blot is shown in (**D**). α-tubulin was used as loading control (51 kDa).

Motor neurons grown in MFCs (DIV6) were treated with BoNT/A (10 nM) for 24 h at 37°C. Importantly, the neurotoxin was added only to the axonal compartment, which is microfluidically isolated from the somatic side. Therefore, appearance of the cleaved fragment of BoNT/A in the latter compartment would imply that full length BoNT/A underwent axonal transport to the cell body and translocated into the cytoplasm. Cells were washed, fixed, permeabilised and stained for BoNT/A-cleaved SNAP25 [17], [19], [20], [34]. Strikingly, the cleaved fragment of SNAP25 was detected both in the axonal and somatic side only in MFCs treated with BoNT/A (BoNT was added only to the axonal compartment)(Figure 8A and B, left panels), but not in control chambers treated with vehicle (Figure 8A and B, right panels), indicating that BoNT/A is not only retrogradely transported in motor neurons but it is also capable of eliciting its catalytic activity following transport. The presence of cleaved SNAP25 in the somatic side of MCFs treated with BoNT/A, but not in control MCFs, was further confirmed by western blot analysis of extracts obtained by pooling the content of three separate chambers (Figure 8C).

To confirm long-distance SNAP25 cleavage in spinal cord motor neurons *in vivo*, we injected BoNT/A into the hind leg muscles of adult rats. Ten days after the delivery of the neurotoxin, lumbar samples of spinal cord were taken and processed for western blot. As shown in Figure 8D, detectable levels of cleaved SNAP25 were found in the spinal cord of BoNT/A-injected animals, but not in sham-treated controls, indicating long-distance neurotoxin action *in vivo*.

### Different kinetics of BoNT/A and TeNT action account for their distinct pathophysiological effects *in vivo*

The demonstration that BoNT/A and TeNT shared a common retrograde transport pathway in motor neurons prompted the key question on why these two neurotoxins display such remarkable differences at pathophysiological level, inducing a flaccid vs. a spastic paralysis, respectively. To directly compare the effects of BoNT/A and TeNT *in vivo*, we chose to test their activity in facial motor neurons projecting to the whisker pad. This model system has two important advantages: i) peripheral neuroparalytic effects can be promptly monitored; and ii) facial motor neurons lack direct proprioceptive and sensory innervation [42], allowing the selective analysis of trafficking events in motor neurons *in vivo*.

**Figure 9:**
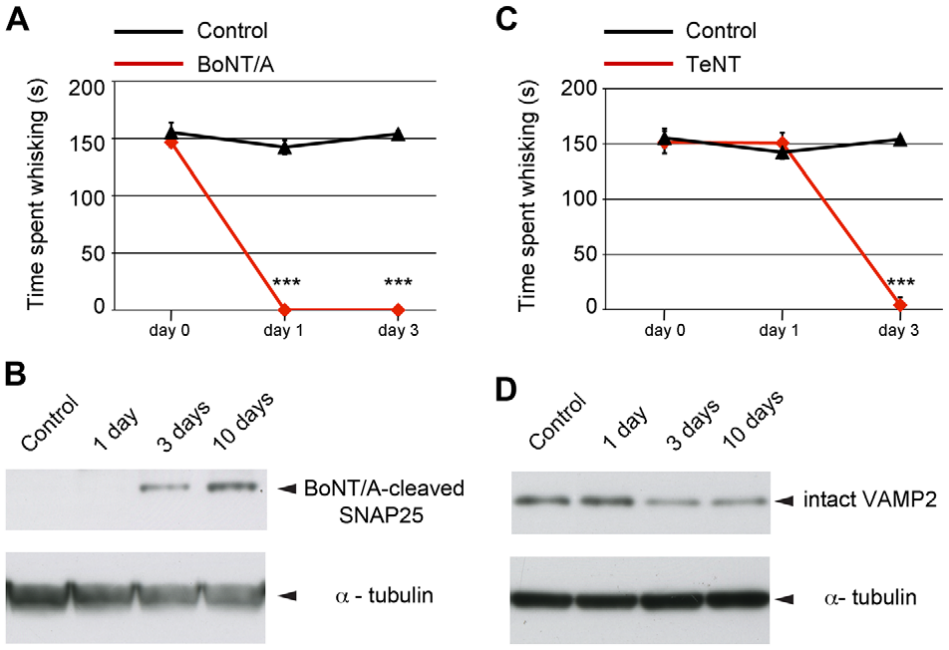
Monitoring peripheral neuroparalytic and central proteolytic effects of BoNT/A and TeNT *in vivo*. BoNT/A (or TeNT) was injected unilaterally into the whisker pad of adult rats. Behavioural analysis of whisking and biochemical detection of proteolytic activity in the brainstem were performed at different times following toxin injection. (**A,C**) Longitudinal assessment of time spent whisking for BoNT/A- or TeNT-treated rats and naïve (control) animals. Note that whisker movements were completely abolished in BoNT/A-treated animals as early as 1 d after injection, while the neuroparalytic effect in TeNT animals was fully apparent at day 3. Quantification reported here is from a representative experiment, which included three animals per group. Data points represent the mean ± standard deviation (SD). Significance was assessed by two-way ANOVA followed by Holm-Sidak test; ***, p<0.001. (**B**) Representative western blot for cleaved SNAP25 (24 kDa) on protein extracts from ipsilateral facial nuclei of BoNT/A-treated rats at different time points after a single toxin injection. SNAP25 cleavage within the facial nucleus (containing motor neuron somas) was detectable starting from day 3 and further increased at day 10. (**D**) Representative immunoblotting for VAMP2 on protein extracts from ipsilateral facial nuclei of TeNT-treated rats at different times after a single toxin injection. Loss of intact VAMP2 (13 kDa) within the facial nucleus was apparent starting from day 3. (**B**, **D**) Results were confirmed in two independent experiments, replicated three times. Representative western blots are shown in (**B**) and (**D**). Each lane represents one animal. Control, naive uninjected rat. Total protein loaded per lane, 50 µg (**B**), 10 µg (**D**). α-tubulin, internal standard, (51 kDa).

We injected BoNT/A (1 nM) or TeNT (3 nM) into the whisker pad of rats and monitored the peripheral effects of the toxins on the behaviour of the animals at different time points. In a separate cohort of animals treated in parallel, we assessed the cleavage of SNAP25 in the ipsilateral brainstem facial nucleus, which contains the cell somas of the motor neurons innervating the whisker pad. On day one following the delivery of the toxin (Figure 9A), we found that BoNT/A completely blocked whisker movements in the treated side. Phenotypically, vibrissae in the injected side were atonic and positioned backward (Movie S5; see an example of a control animal in Movie S6). However, BoNT/A-cleaved SNAP25 in the facial nucleus was only clearly detectable from day three (Figure 9B), indicating a temporal shift between peripheral and central action of BoNT/A. Interestingly, we observed a build up of central cleaved SNAP25 over time (Figure 9B), suggesting progressive cumulative effects. In striking contrast, TeNT action followed a different time course. Both the peripheral paralysis and central VAMP2 proteolysis (detected as decreased levels of intact VAMP2 in treated animals) occurred with very similar kinetics and were overt at day three (Figure 9C and D). At this time point, whiskers appeared rigid and immobile, protruding at a right angle from the snout (Movie S7). This is consistent with a spastic paralysis, indicating TeNT action on inhibitory circuits after retrograde trafficking in brainstem motor neurons.

These results demonstrate that, in the case of BoNT/A, the peripheral action precedes the central one, whilst their kinetics overlap for TeNT. Thus, flaccid paralysis of injected muscle is maintained by the fast and robust blockade of acetylcholine release from peripheral nerve terminals, whilst neural circuits serving other (e.g. antagonistic) muscles can be significantly affected by centrally active BoNT/A.

## Discussion

The textbook explanation of the differences between the clinical symptoms of tetanus and botulism is based on the distinct fates of TeNT and BoNTs once internalised in motor neurons [4], [6]. Recent *in vivo* and *in vitro* evidence are now challenging this paradigm, suggesting that BoNTs might undergo long-distance axonal transport, especially at high doses. Early experiments with radiolabelled full length BoNT/A showed that the toxin is transferred to the ventral roots and adjacent spinal cord segments upon intramuscular injection in the cat gastrocnemius [43], [44]. Similarly, Black and Dolly [45] observed radiolabelled BoNT/A within the axoplasm of myelinated axons after its peripheral injection in mice. A dose-dependent retrograde transport of BoNT/A in brainstem motor neurons was also shown by electrophysiological and ultrastructural experiments in cats [46], [47]. In compartmentalised cultures of rat sympathetic neurons, BoNT/A moves retrogradely into cell bodies when applied at high concentrations into the distal compartments [18]. Finally, Antonucci *et al.* provided evidence for retrograde transport and transcytosis of BoNT/A in rat facial motor neurons after its injection into the whisker pad [20]. However, retrograde trafficking of BoNTs has been inferred mainly indirectly, i.e. by observing the appearance of radioactivity or BoNT-cleaved substrates away from the site of administration. Thus, the kinetics and intracellular pathways used by BoNTs for their long-range transport remains unclear.

Our work was designed to fill this gap, using differentiated motor neurons and both the binding domain and the full length forms of BoNT/A and BoNT/E. H_C_A and H_C_E were found to have comparable uptake and transport properties to the full length neurotoxins, implying that the H_C_ domain carries the minimum determinant(s) for long-range transport [41], [48]. We found that the uptake of H_C_A and H_C_E in motor neurons is enhanced under depolarisation, a condition that stimulates BoNT endocytosis into central neurons [27], [30], [49], [50]. Thus, synaptic vesicle recycling, which is increased under depolarising conditions, plays an important role in toxin uptake in motor neurons. However, this is unlikely to be the only internalisation route exploited by BoNTs to enter these neurons. Pre-treatment with BoNT/D, which blocks exocytosis by selectively cleaving VAMP [6], does not completely prevent internalisation of H_C_A and H_C_E in these cells. Thus, an internalisation route independent of synaptic vesicle recycling should be considered for the entry of BoNTs in motor neurons. Such a pathway may involve the small fraction of SV2 that resides at steady state on the plasma membrane, together with other synaptic vesicle proteins [51], [52]. However, the incomplete colocalisation of SV2A and C with H_C_s suggests the involvement of additional endocytic route(s) for BoNT/A and/E uptake in motor neurons.

Our time-lapse analyses in motor neurons in *mass* culture and in MFCs demonstrated that BoNT/A and BoNT/E, and their H_C_ domains are retrogradely transported in neurons. These results provide direct evidence that at least a fraction of internalised BoNT is capable of fast long-range trafficking, consistent with previous data [18], [20], [43], [44], [46], [47]. Determining the proportion of BoNTs entering the local (synaptic vesicle-based) versus distal (retrograde endosome-based) trafficking pathways is potentially very important to address the balance between peripheral and central effects of these neurotoxins. Although we cannot yet provide conclusive data to address this question, the quantification of the uptake of HcA and HcE under resting or depolarising conditions indicates that about 50% of these fragments are internalised in a stimulation-independent manner (Figure 2C and 2D). Only a fraction of this pool is targeted to the axonal retrograde pathway, since direct comparison with H_C_T indicates that H_C_A and H_C_E are significantly less efficient in being recruited to this route, as exemplified by their higher proportion of stationary carriers and their overall lower frequency of transport (about 20–30% of H_C_T). In addition, movement of BoNT/E was less continuous compared to BoNT/A, as shown by the occurrence of stops and transitory changes in direction of these axonal endosomes. Based on these considerations, the overall proportion of distally targeted H_C_A would be between 10 and 15%, and even less for H_C_E. These values are in good agreement with the proportion of BoNT/A targeted to long-range axonal transport in the visual system (5 to 10%; LR and MC, unpublished results).

The high degree of colocalisation observed during uptake and transport indicates that H_C_A and H_C_E share the same organelles once internalised into neurons. A partial colocalisation of H_C_A- and H_C_T-positive axonal puncta was also apparent during transport. Since H_C_T and TeNT use a transport pathway shared with neurotrophins and their receptors [21], [23], this result suggests that the same pathway is used by H_C_A and H_C_E for long-distance trafficking. This conclusion has been confirmed by our findings that p75^NTR^ and H_C_A enter the same axonal compartment in motor neurons. Retrogradely-moving BoNT-positive carriers display negligible colocalisation with Lysotracker, a marker of acidic vesicles. This is particularly important, since acidic pH is known to induce the translocation of the L chain of BoNTs into the cytosol [41], [53]. Protection of BoNT-containing carriers from a drop in pH during transport may be key in order to entrap the neurotoxin into the lumen of the transport organelle during its transfer to the soma. Catalytically intact BoNT may eventually reach suitable release sites and become available for transcytosis to connected neurons in the network [19], [20], [34], as shown for TeNT [16], [54], [55].

Interfering with the acidification of the early endosomal compartment responsible for the synaptic uptake of H_C_A and H_C_E is a possible strategy to re-direct these fragments to the axonal retrograde pathway. However, this approach could not be pursued in our experimental system, since preventing endosome acidification by inhibiting the vATPase with concanamycin A or bafilomycin A impairs very early events in the sorting of H_C_T and halts its recruitment to retrogradely-transported signalling endosomes [22]. Moreover, a functional vATPase is required for other endocytic mechanisms linked to regulated secretion, such as the retrieval of secretory granule proteins stranded on the plasma membrane, which may be exploited by BoNT/A and BoNT/E to enter motor neurons upon binding to SV2 [56].

The similarity of trafficking mechanisms exploited by TeNT and BoNTs needs to be reconciled with the vastly different clinical symptoms of botulism and tetanus. Although alternative hypotheses have been previously suggested [57], our *in vivo* studies on facial motor neurons offer a sound explanation. Similar doses of BoNT/A and TeNT resulted in clearly distinct kinetics of the peripheral neuroparalytic effects, with TeNT inducing a delayed blockade of whisker movements (day three) compared to BoNT/A (day one). Conversely, the central proteolytic action displays a very similar onset for the two neurotoxins, starting after three days from injection. Whereas for TeNT the peripheral and central effects coincide, for BoNT/A the central effect lags behind the onset of peripheral symptoms. Consequently, the blockade of acetylcholine release at the neuromuscular junction “masks” any alteration in motor neuron firing caused by the central action of BoNT/A, thus leaving the injected muscle persistently flaccid. However, functional long-term consequences of BoNT/A acting at the level of central circuits [34], should not be overlooked. In this regard, any neurotoxin-induced modification in the strength of spinal cord synapses impinging onto motor neurons is bound to alterations in their output that in turn may affect firing of inhibitory interneurons, such as Renshaw cells [58], [59], with a substantial impact on antagonistic and/or synergistic muscles. This may occur via the widespread projections of motor neuron recurrent collaterals to Renshaw cells impinging on motor nuclei supplying muscles acting at the same joint or trans-joint [60].

Although our results provide a rationale of the different neuroparalytic effects of TeNT and BoNTs, much more work is required to quantitatively assess the targeting of BoNT/A and BoNT/E to distal sites. Therefore, a major aim of future experiments is determining the dose dependence of the effects of these neurotoxins at synaptic sites and in the soma both *in vitro* and *in vivo*, a strategy that would indirectly address the proportion of BoNT/A, BoNT/E and TeNT taken up by the acidic pathway. This analysis is however complicated by the methodology used for the detection of the activity of these neurotoxins, which is based on antibodies recognizing the cleaved SNARE proteins. Indeed, this approach is highly dependent on variations in the enzymatic activity of the L chains of different serotypes, their efficiency of translocation into the cytoplasm, their post-translational modifications and the relative intracellular stability of both the L chain and the cleaved substrates, parameters for which our present understanding is very limited [6], [41], [61], [62].

**Figure 10:**
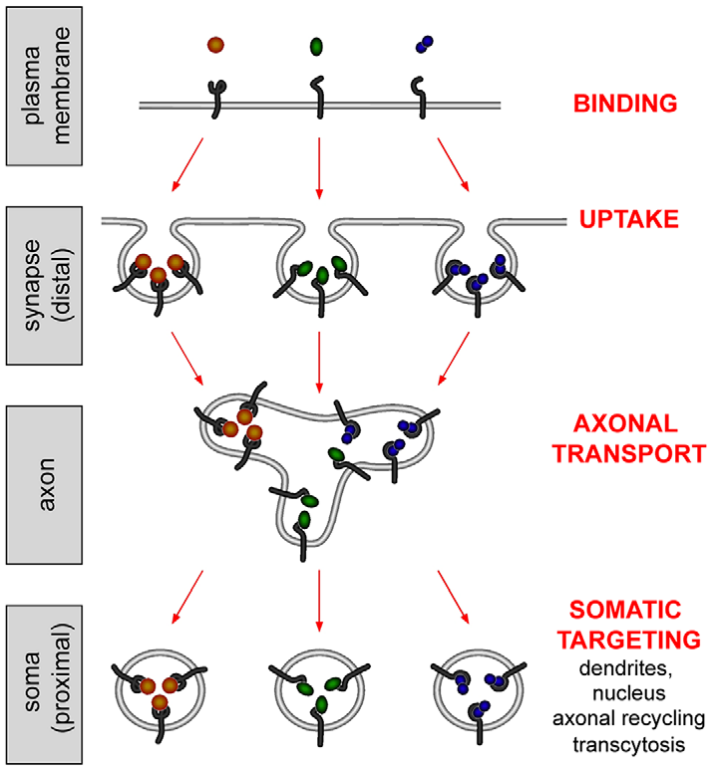
Biogenesis of axonal retrograde transport carriers and their sorting in the soma. Pathogens and virulence factors have been shown to exploit the axonal retrograde transport pathway to gain access to the central nervous system. Although different internalisation routes are used to enter neurons by different pathogens, pathogen-receptor complexes are sorted to communal carriers to undergo long-range axonal transport to reach the central nervous system. These carriers, which have been shown to contain BoNTs and TeNT, poliovirus, canine adenovirus 2, cholera toxin B subunit and their cellular receptors, are non acidic and non degradative. Cargoes transported by these organelles undergo differential sorting upon arrival in the soma, where they are then directed towards their final destination. See text for further details.

Several pathogens and virulence factors have been shown to exploit axonal retrograde transport pathways to spread into the central nervous system. We have recently shown that poliovirus [63] and canine adenovirus serotype 2 (CAV2) [40] enter the same transport carriers used by TeNT and BoNTs together with their physiological receptors. This result is surprising since these pathogens as well as several endogenous cargoes are taken up by neurons using different endocytic mechanisms. For example, even though the B subunit of cholera toxin (CTB) binds to the ganglioside GM1 and is internalised via a clathrin-independent route [64], it is co-transported together with H_C_T and p75^NTR^, which are taken up by clathrin-mediated endocytosis [39], [64]. Therefore, mechanisms operate at distal sites of neurons (e.g. the neuromuscular junction) to sort these diverse endosomal cargoes to common non-degradative organelles, which are then recruited to a long-range axonal transport route. This whole sequence of events serves to translocate endogenous ligands, pathogens and virulence factors from the periphery of motor and sensory neurons to the central nervous system. It is plausible that these transport carriers undergo another sorting step once they arrive in the soma, a process that would provide a novel regulatory mechanism in communicating information from nerve terminals to the cell body (Figure 10). The presence of fewer types of retrograde transport organelles than anticipated could have profound effects on axonal homeostasis and the regulation of overall cargo flow. A limited number of carrier types is likely to streamline the mechanisms ensuring motor recruitment and cargo transfer, which would occur mainly at hubs positioned at distal nerve terminals and in the soma, thus simplifying the control of membrane flow in the axon. The presence of multiple receptors and ligands in these transport carriers would also enhance their plasticity in terms of signalling potential. Thus, the signal(s) generated by a single receptor/ligand complex could vary in amplitude, frequency and outcome based on the presence of other cargoes for a given retrogradely-transported organelle. Uncovering the determinants of these transport and sorting mechanisms will provide new insights on how long range communication is regulated and will identify new targets for the control of trafficking of pathogens in the nervous system.

Whereas this data indicates a robust retrograde transport of H_C_E and BoNT/E in cultured motor neurons, previous experiments based on unilateral BoNT/E delivery into the rodent brain failed to find evidence for propagation of the effects induced by BoNT/E to the contralateral hemisphere [20], [65], [66]. There are several possible reasons for this discrepancy, including the different experimental systems (spinal cord motor neurons in culture vs. central neurons *in vivo*). Importantly, the *in vivo* experiments used cleavage of SNAP25 as a detection method for long-range BoNT/E trafficking, which requires not only axonal transport, but appropriate somatic sorting of these carriers, transcytosis and entry into a compartment which enables the translocation of the L chain into the cytoplasm. Previous work with neurotrophins has shown that some retrogradely transported cargoes, such as NGF, undergo lysosomal degradation, whilst others (e.g. BDNF) are released at synaptic sites, where they can affect second-order neurons [16], [54]. It is conceivable that BoNT/A and BoNT/E might undergo differential sorting events at the cell soma, which could impact on their ability to undergo transcytosis. Another non-mutually exclusive possibility is the preferential degradation of the L chain of BoNT/E due to ubiquitination and proteasome targeting [62]. This is particularly relevant to the *in vivo* system, where BoNT/E trafficking has been examined in long-distance projecting neurons [20], [65], [66]. In this case, the rapid degradation of BoNT/E would not allow the accumulation of detectable amounts of truncated SNAP25 at distal sites. Conversely, the prolonged catalytic activity of BoNT/A [20], [62], [67], [68] would enable the occurrence of long-distance effects and the detection of truncated SNAP25.

Our demonstration of retrograde transport of BoNT/A in spinal cord motor neurons may have implications for the analysis of the central effects of this neurotoxin in the clinic. BoNT/A and B are used for the treatment of many human pathologies characterised by hyperactivity of nerve terminals and hypersecretory syndromes [7], [10]. The clinical benefits depend mainly on a localised neuromuscular blockade, but there is substantial evidence for central effects of BoNT/A, which could contribute to the overall therapeutic efficacy [69]–[71]. These central effects may depend either on neuronal plasticity, or on a direct BoNT/A activity on central synapses. Our data provide evidence in favour of such direct action.

The ability of H_C_A and H_C_E to undergo retrograde trafficking holds promise for the development of novel drug delivery vehicles for the targeting of therapeutics to the central nervous system. A similar approach was previously applied to H_C_T, which has been exploited for the delivery of various molecules to central neurons [72], [73]. The translation of H_C_T derivatives to clinical practice is not straightforward however, due to the presence of circulating antibodies directed against TeNT in most individuals as a result of the widespread vaccination against tetanus in industrialised countries. In this context, the implementation of BoNT H_C_-based carriers might overcome this limitation and provide a novel class of drug delivery systems.

## Materials and Methods

### Ethics statement

All experiments were carried out following the guidelines of the Cancer Research UK genetic manipulation and Ethic Committees and in accordance with the European Community Council Directive of November 24, 1986 (86/609/EEC). *In vivo* experiments were approved by the Italian Ministry of Health. Animal work was carried out under licence from the UK Home Office in accordance with the Animals (Scientific Procedures) Act 1986.

### Reagents

Reagents were from Sigma unless stated otherwise. Restriction enzymes were from New England Biolabs. Lysotracker RedDND-99, AlexaFluor maleimides and AlexaFluor-conjugated secondary antibodies were from Life Technologies. Taq polymerase, bacterial media and Hank's buffer (20 nM HEPES-NaOH pH 7.4, 0.44 mM KH_2_PO_4_ 0.42 mM NaH_2_PO_4_, 5.36 mM KCl, 136 mM NaCl, 0.81 mM MgSO_4_, 1.26 mM CaCl_2_, 6.1 mM glucose) were provided by Cancer Research UK Central Services.

H_C_A (residues 860–1296) and H_C_E (residues 820–1252) were expressed as GST-fusion proteins in *E. coli* BL21 [74] and after purification, dialyzed against 20 mM HEPES-NaOH pH 7.4, 1 M NaCl. H_C_T was prepared as previously described [22]. In selected experiments, a shorter version of H_C_T (H_C_T441, residues 875–1315) fused to an improved cysteine-rich tag [25], which has a longer shelf life, was used. BoNT/A was prepared and tested as previously described [19], [20], [34], [75], whilst TeNT was from Lubio [76]. Purified full length BoNTs were labelled with AlexaFluor488 (green) or 568 (red) according to the manufacturer's instructions. The moles of dye per mole of BoNT averaged 6.0. Fluorescent H_C_s and BoNTs were dialysed against HEPES-NaOH 10 mM pH 7.4, 150 mM NaCl before use.

### Neuronal cultures

Spinal cord motor neurons were prepared from 14.5 day old rat embryos (Sprague-Dawley, Charles River) [77] or 13.5 day old mouse embryos [78], [79], and plated onto poly-L-ornithine and laminin-coated glass coverslips, MatTek dishes or MFCs, and cultured at 37°C and 7.5% CO_2_. Motor neurons were used for experiments starting from day *in vitro* 5 (DIV5) until DIV10. For binding studies, motor neurons were pre-cooled on ice, washed with 0.2% BSA in Hanks' buffer, and incubated with AlexaFluor-labelled H_C_s (7.5–15 nM) or full length BoNTs (15 nM) for 15 min. MNs were then washed in PBS and fixed in 4% paraformaldehyde (PFA) containing 20% sucrose. For endocytosis assays, motor neurons were incubated at 37°C with fluorescent H_C_s (7.5–15 nM) or full length BoNTs (15 nM) for 30 min in resting (NaCl 137 mM, KCl 5 mM, MgCl_2_ 1 mM, CaCl_2_ 2.5 mM, glucose 10 mM, HEPES-NaOH 5 mM, pH 7.4) or depolarising (NaCl 80 mM, KCl 60 mM, MgCl_2_ 1 mM, CaCl_2_ 2.5 mM, glucose 10 mM, HEPES-NaOH 5 mM, pH 7.4) conditions. Neurons were then cooled on ice, washed with acidic buffer (100 mM citrate-NaOH, 140 mM NaCl, pH 2.0) for 5 min at room temperature in order to remove the probe still bound to the cell surface, washed with PBS and fixed. In selected cases, motor neurons (DIV6) were pre-treated with 2 nM BoNT/D (Wako) for 22 h at 37°C, whereas controls were left untreated.

To assess the activity of BoNT/A *in vitro*, neurons were incubated with 10 nM BoNT/A added only to the axonal side of MFCs for 24 h at 37°C. A higher volume of media was added to the cell body compartment to avoid passive diffusion. For western blot analysis, neurons were cooled on ice and the cell bodies were harvested in RIPA buffer (10 mM Tris-HCl, pH 7.5, 150 mM NaCl, 0.1% SDS, 1% Triton X-100, 5 mM EDTA) containing protease and phosphatase inhibitors (Thermo Scientific).

To assess the activity of BoNT/A *in vivo*, proteins were extracted with lysis buffer (1% Triton X-100, 10% glycerol, 20 mM Tris-HCl, pH 7.5, 150 mM NaCl, 10 mM EDTA, 0.1 mM Na_3_VO_4_, 1 µg/ml leupeptin, 1 µg/ml aprotinin, and 1 mM PMSF). Proteins (total loading: 10 µg for VAMP2 and 50 µg for cleaved-SNAP25) were separated on 4–12% pre-cast gels (Life Technologies), transferred onto PVDF membranes, blocked and then incubated with primary antibodies overnight at 4°C (anti-BoNT/A-cleaved SNAP25 [17], [19], [20], [34], 1∶500; anti-VAMP2, 1∶15,000, Synaptic Systems; anti-βIII tubulin (Tuj1), 1∶1,000, Millipore; anti-α-tubulin, 1∶10,000, Sigma). Secondary horseradish peroxidase-conjugated antibodies (Jackson ImmunoResearch) were used at 1∶1,000 dilution for 1 h at room temperature, and the resulting signal detected by enhanced chemiluminescence (GE healthcare).

MFCs were produced using established methods [35], [36]. Polydimethylsiloxane (Dow Corning) inserts were sterilised and fixed to 50 mm glass-bottomed WillCo dishes (IntraCel) using plasma cleaning. MFCs were blocked with 0.8% BSA in PBS overnight at 37°C and then coated with poly-L-ornithine and laminin. Motor neurons were plated in the somatic compartment of the MFC and left to adhere before the full medium was applied. Experiments were performed between DIV7-10 when axons crossed the microgrooves and reached the distal side of MFCs.

### Immunofluorescence

Motor neurons were fixed in 4% PFA containing 20% sucrose in PBS for 15 min at room temperature, washed in PBS and then permeabilised with 0.1% Triton X-100 in PBS for 5 min. Cells were subsequently treated with blocking buffer (10% heat-inactivated horse serum, 2% BSA in PBS) for at least 30 min at room temperature. Samples were then incubated for 30 min with the following primary antibodies: anti-SV2A (R16; sc11939; Santa Cruz, and SYSY119002; Synaptic System), anti-SV2B (SYSY119102; Synaptic System) anti-SV2C (P20; sc11946; Santa Cruz and an isoform-specific polyclonal antibody developed by T. Südhof [80]), anti-VAMP2 (SYSY104211; Synaptic Systems), anti-bIII tubulin (Tuj1; MMS435P, Covance), anti-p75^NTR^ [39], anti-BoNT/A-cleaved SNAP25 [17], [19], [20], [34], followed by the appropriate fluorescently-labelled secondary antibody for 30 min at room temperature. Coverslips were washed with three times in PBS and mounted on glass slides in Moviol-488 (Harco).

Colocalisation was quantified by applying a mask corresponding to the whole neuronal network obtained using an antibody against βIII-tubulin (Tuj1). Fluorescence intensity of H_C_s and SV2 isoforms was determined in resting and stimulating conditions in the area of the mask using ImageJ. The level of colocalisation was assessed by measuring the Mander's coefficient on randomly chosen fields. Statistical significance was calculated using Mann-Whitney test.

### Axonal retrograde transport assays

Motor neurons plated onto MatTek glass-bottom dishes or onto MFCs were incubated with fluorescent H_C_A and E as described above. In selected experiments, motor neurons were co-incubated with 50 nM LysoTracker or with an antibody against the extracellular domain of p75^NTR^ receptor (1∶1,000 dilution). After incubation for 30 min at 37°C under resting or stimulating conditions, cells were washed with E4 imaging medium containing 30 mM HEPES-NaOH, pH 7.4 and imaged by time-lapse confocal microscopy at 37°C. Images were acquired every 4 s over a total of up to 200 frames using a Zeiss LSM 510 confocal microscope equipped with a Zeiss 63X, Phase 3 Plan Apochromat oil-immersion objective and controlled by Zeiss LSM 510 software.

Carriers were tracked manually using Motion Analysis Software (Kinetic Imaging). Single-movements between two consecutive frames were measured to determine the speed of the carrier. Only moving carriers that could be followed for a minimum of four consecutive frames were analysed, and tracking was stopped when the organelle went out of focus or stopped for the remaining observation time. The distance covered by a carrier between two consecutive frames, termed single movement, was used to determine its instantaneous speed. A double-positive compartment was defined on the basis of the following criteria: i) the carrier was labelled in two different channels; ii) the morphology of the carrier was very similar in the two channels; and iii) its speed and direction was identical in the two channels for at least 4 time points in a time-series. Statistical analysis and curve fitting were performed using Kaleidagraph (Synergy Software). Kymographs were generated using MetaMorph (Molecular Devices) after rotation of the image stack to align the neuronal process vertically. Horizontal single line-scans through the thickness of each process were plotted sequentially for every frame in the time series.

Colocalisation of double-positive carriers was quantified by MetaMorph using “manually-count objects” options. For this, all H_C_-positive carriers were manually marked and automatically counted, then the other channel (i.e. Lysotracker) was overlaid and double-positive carriers were highlighted and counted. Student t-test was performed using Kaleidagraph.

### *In vivo* experiments

Adult Long-Evans rats (35 in total) were kept on a 12 h light/dark cycle and had access to food and water ad libitum. Animals were anaesthetized with isoflurane and injections of BoNT/A (1 nM, 0.5 µl; n = 12) or TeNT (3 nM, 0.5 µl, n = 11) were performed with a microsyringe on the right side of the snout at the centre of the whisker pad (i.e., between rows B and C of the vibrissae) [20], [81], [82]. Three naive control animals were also included. Brains were dissected out at 1, 3 or 10 d and 500 µm thick coronal sections were cut through the brainstem with a microtome (Leica). The facial nucleus ipsilateral to the injection site was microdissected and immediately frozen.

For the behavioural analysis, whisker movements were monitored for each animal before injection (baseline), and 1 and 3 d following toxin delivery (n = 6). Control naïve rats (n = 3) were monitored with an identical schedule. Each rat was placed in a clear plexiglas cylinder [83] and filmed for 3 minutes. Total time spent during whisking (injected side) was calculated offline for each movie. Statistical significance was calculated with two-way ANOVA followed by Holm-Sidak test.

Another group of animals were anaesthetized with isoflurane and a small skin incision was performed to expose the tibialis anterior and the gastrocnemius muscles [84]. Muscles were injected with BoNT/A (1 nM, 1 µl, n = 4 rats). After 10 d spinal cords were dissected and lumbar segments were taken and immediately frozen for western blotting.

## Supporting Information

Figure S1 **Quantification of the colocalisation of H_C_A and HcE with SV2 isoforms A and C under resting and depolarising conditions.** H_C_A does not show any preference between SV2A and C in primary rat motor neurons (**A**). In contrast, H_C_E colocalises significantly more with SV2C in both resting and depolarising conditions (Mann-Whitney test; **, p<0.01, ***, p<0.001) (**B**). However, the colocalisation is not complete for both H_C_s and does not change upon depolarisation. The study reported in this figure was performed using at least two independent primary motor neuron cultures. At least ten fields were analysed for each conditions. Quantification reported here is from a representative experiment.
(TIF)

Figure S2 **Full length BoNT/A and BoNT/E are internalised in motor neurons.** Motor neurons were incubated with 30 nM AlexaFluor488-BoNT/A (**A**) or 30 nM AlexaFluor555-BoNT/E (**B**) for 30 min at 37°C, either under resting conditions or after stimulation (60 mM KCl). Motor neurons were placed on ice, acid washed, fixed, and stained for SV2C. BoNT uptake slightly increases under stimulating conditions, as well as the colocalisation between BoNTs and SV2C. The study reported in this figure was performed using a primary motor neuron culture and repeated twice. Shown are representative images for each condition. Scale bars, 20 µm.
(TIF)

Figure S3 **H_C_A and H_C_T display extensive colocalisation in motor neurons.** Motor neurons were incubated with 15 nM H_C_A and 40 nM AlexaFluor555-TeNT H_C_ (H_C_T) for 30 min at 37°C, either under resting (5 mM KCl) or stimulating conditions (60 mM KCl). Cells were then placed on ice, acid washed and fixed. An extensive colocalisation between H_C_A and H_C_T was observed after internalisation in motor neurons. Inset: high magnification of the indicated areas. This analysis was performed using two independent primary motor neuron cultures. Shown are representative images for each condition. Scale bars, 20 µm.
(TIF)

Movie S1 **H_C_A undergoes fast retrograde axonal transport in primary motor neurons in MFC.** Motor neurons grown in MFC were incubated with fluorescent H_C_A for 30 min at 37°C in resting conditions, then washed and imaged by time-lapse confocal microscopy. The toxin was added exclusively to the axonal side. Cell body is out of view on the right. Frames were taken every 3 s and the movie plays at 5 frames/s. This movie is a representative example of experiments performed on two independent primary motor neuron cultures, replicated three times.
(MP4)

Movie S2 **H_C_A shares fast retrogradely transported organelles with the neurotrophin receptor p75^NTR^.** Motor neurons seeded in MFC were incubated for 30 min at 37°C with fluorescent H_C_A (red) and a fluorescently-labeled antibody against p75^NTR^ (green) in resting conditions, then washed and imaged. Both H_C_A and the antibody have been added to the axonal side only of the MFC. The two channels are merged in the movie. Yellow structures indicate double positive organelles. Cell body is out of view on the right. Frames were taken every 3 s and played at 5 frames/s. This movie is a representative example of experiments performed on two independent primary motor neuron cultures.
(MP4)

Movie S3 **H_C_A undergoes fast retrograde transport in non acidic organelles.** Motor neurons cultures were incubated with fluorescent H_C_A (green) and Lysotracker (red) for 30 min at 37°C under depolarising conditions (60 mM KCl). Cells were then washed and imaged. H_C_A undergo fast retrograde transport (from left to right) in non acidic organelles as demonstrated by the almost total lack of colocalisation with Lysotracker. Cell body is out of view on the right. Frames were taken every 4 s and played at 5 frames/s. This movie is a representative example of experiments performed on two independent primary motor neuron cultures, replicated twice.
(MP4)

Movie S4 **H_C_E undergoes fast retrograde transport in non acidic organelles.** Motor neurons cultures were incubated with fluorescent H_C_E (green) and Lysotracker (red) for 30 min at 37°C under depolarising conditions (60 mM KCl). Cells were then washed and imaged. H_C_E is retrogradely transported in organelles lacking Lysotracker. As discussed in the text, some of the carriers containing H_C_E (green) show a discontinuous transport in the retrograde direction (from left to right), whereas some others undergo anterograde transport. Cell body is out of view on the right. Frames were taken every 4 s and played at 5 frames/s. This movie is a representative example of experiments performed on two independent primary motor neuron cultures, replicated twice.
(MP4)

Movie S5 **Whisking behaviour in the Schallert cylinder of a representative rat injected with BoNT/A (1 nM, 0.5 µl) into the right whisker pad 1 d earlier.** Note that vibrissae on the right side of the snout are atonic and positioned backward, indicating a flaccid paralysis due to the local effect of the toxin. A similar behaviour was observed in all animals of the group.
(MOV)

Movie S6 **Representative whisking behaviour in the Schallert cylinder of a control, uninjected rat.**
(MOV)

Movie S7 **Whisking behaviour in the Schallert cylinder of a representative rat injected with TeNT (3 nM, 0.5 µl) into the right whisker pad 3 d earlier.** Note immobile, rigid vibrissae on the right side of the snout indicating a spastic paralysis. A similar behaviour was observed in all animals of the group.
(MOV)
