# Supplementary figures and images for: Botulinum Neurotoxins A and E Undergo Retrograde Axonal Transport in Primary Motor Neurons
Source: PLoS Pathog. 2012 Dec 27;8(12):e1003087. doi: 10.1371/journal.ppat.1003087 (PMC3531519; doi:10.1371/journal.ppat.1003087)

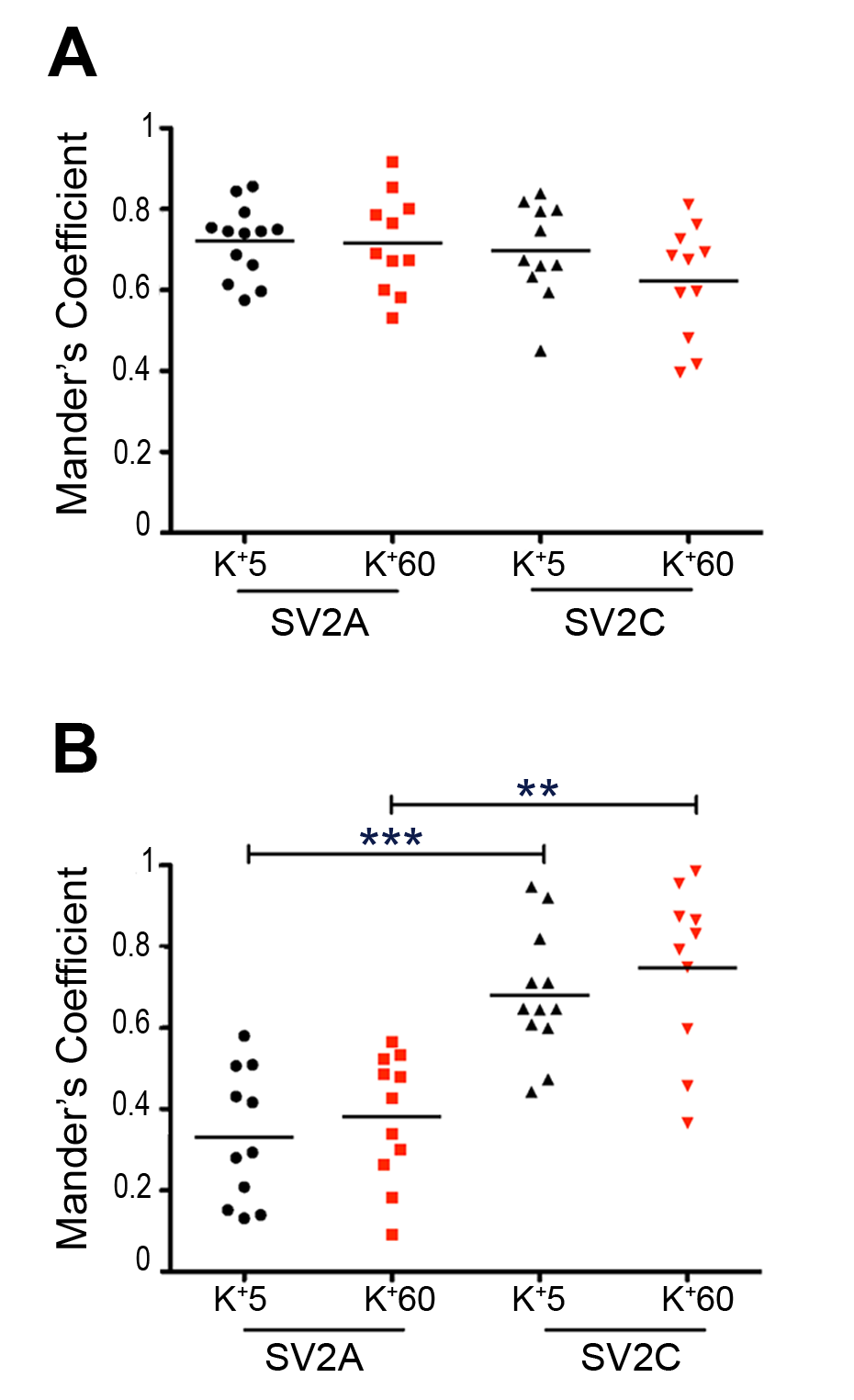

Supplement: Figure S1 — Quantification of the colocalisation of HCA and HcE with SV2 isoforms A and C under resting and depolarising conditions. HCA does not show any preference between SV2A and C in primary rat motor neurons (A). In contrast, HCE colocalises significantly more with SV2C in both resting and depolarising conditions (Mann-Whitney test; **, p<0.01, ***, p<0.001) (B). However, the colocalisation is not complete for both HCs and does not change upon depolarisation. The study reported in this figure was performed using at least two independent primary motor neuron cultures. At least ten fields were analysed for each conditions. Quantification reported here is from a representative experiment. (TIF) [file ppat.1003087.s001.tif]

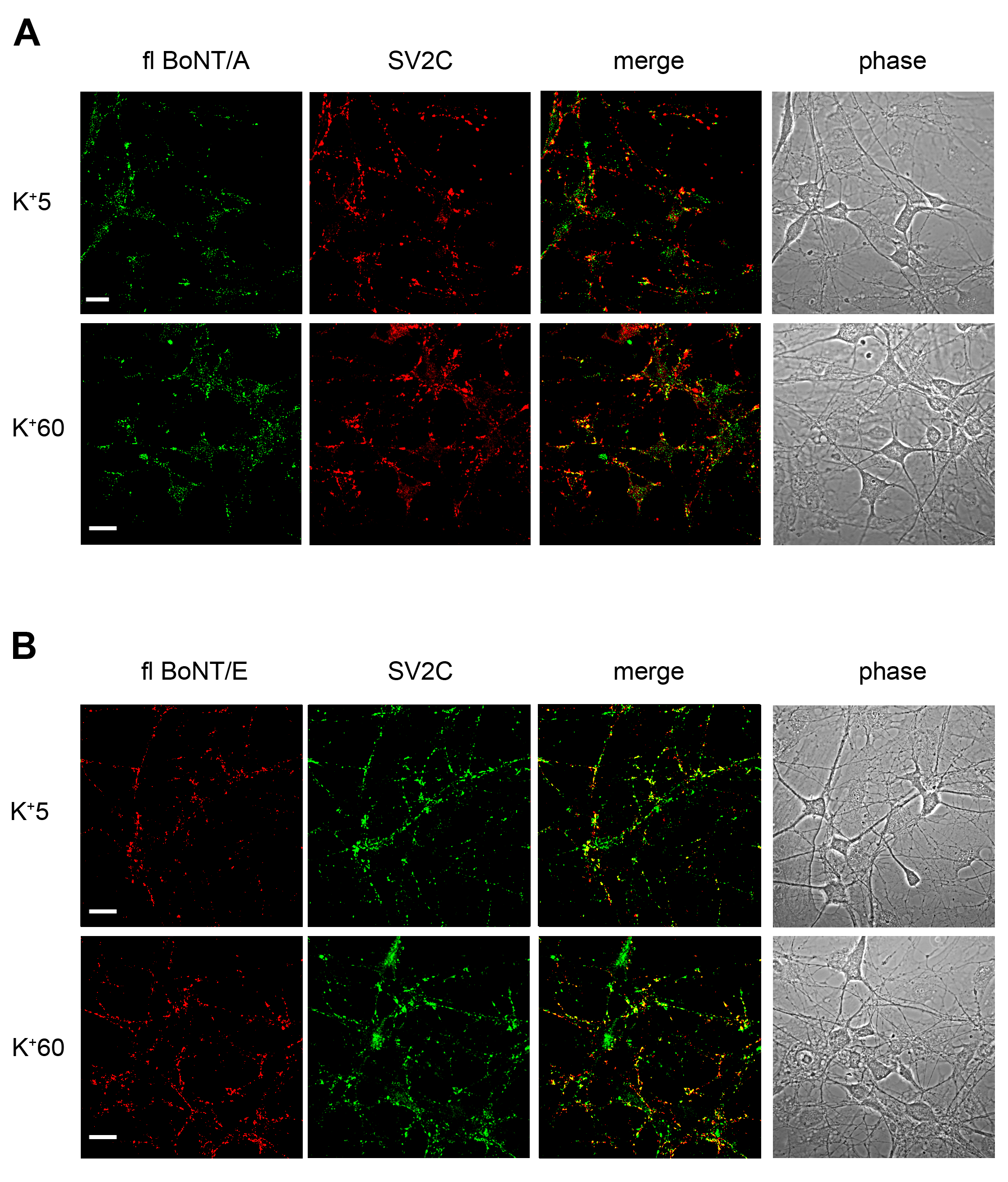

Supplement: Figure S2 — Full length BoNT/A and BoNT/E are internalised in motor neurons. Motor neurons were incubated with 30 nM AlexaFluor488-BoNT/A (A) or 30 nM AlexaFluor555-BoNT/E (B) for 30 min at 37°C, either under resting conditions or after stimulation (60 mM KCl). Motor neurons were placed on ice, acid washed, fixed, and stained for SV2C. BoNT uptake slightly increases under stimulating conditions, as well as the colocalisation between BoNTs and SV2C. The study reported in this figure was performed using a primary motor neuron culture and repeated twice. Shown are representative images for each condition. Scale bars, 20 µm. (TIF) [file ppat.1003087.s002.tif]

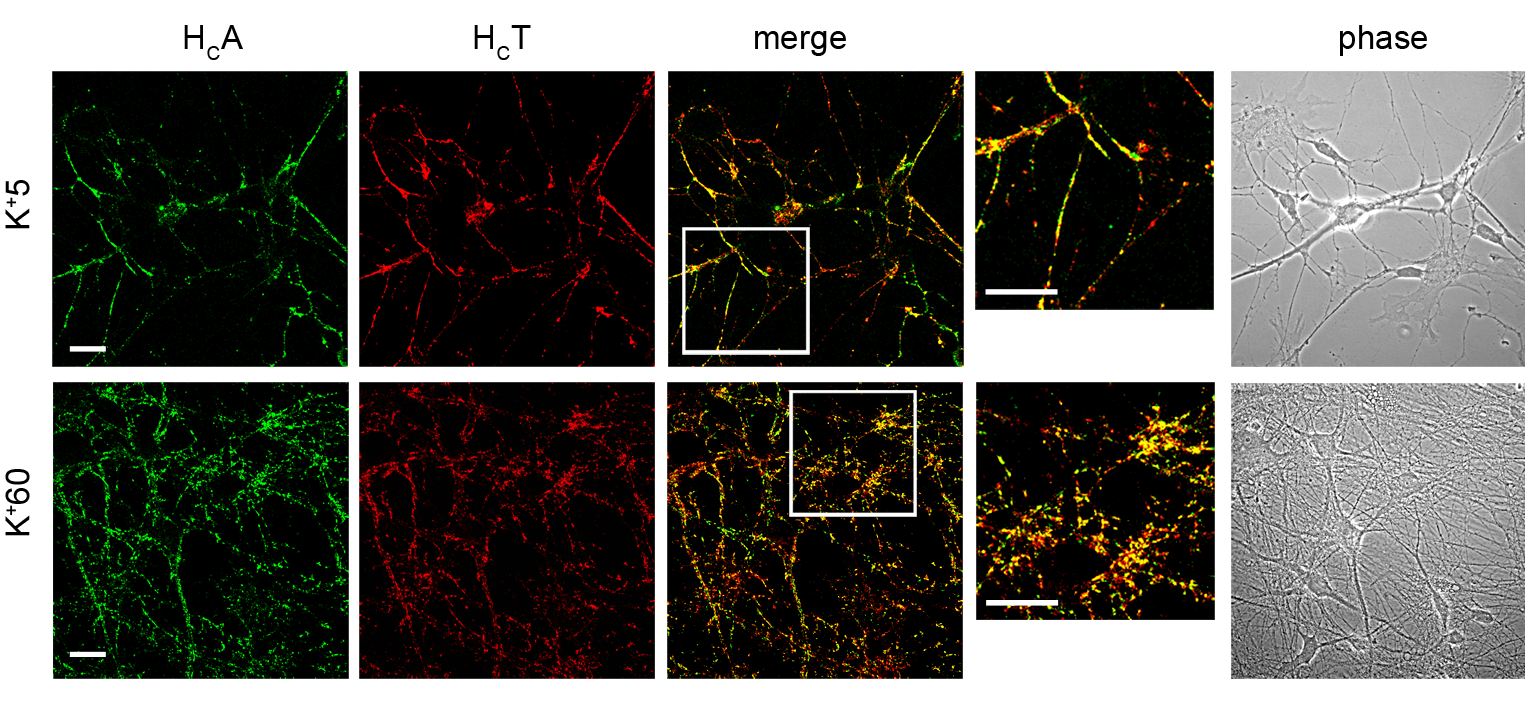

Supplement: Figure S3 — HCA and HCT display extensive colocalisation in motor neurons. Motor neurons were incubated with 15 nM HCA and 40 nM AlexaFluor555-TeNT HC (HCT) for 30 min at 37°C, either under resting (5 mM KCl) or stimulating conditions (60 mM KCl). Cells were then placed on ice, acid washed and fixed. An extensive colocalisation between HCA and HCT was observed after internalisation in motor neurons. Inset: high magnification of the indicated areas. This analysis was performed using two independent primary motor neuron cultures. Shown are representative images for each condition. Scale bars, 20 µm. (TIF) [file ppat.1003087.s003.tif]
